# Insights from the Absorption Coefficient for the Development of Polarizable (Multipole) Force Fields

**DOI:** 10.3390/molecules30142941

**Published:** 2025-07-11

**Authors:** Marion Sappl, András Szabadi, Philipp Honegger, Franziska König, Othmar Steinhauser, Christian Schröder

**Affiliations:** 1 Department of Computational Biological Chemistry, Faculty of Chemistry, University of Vienna, Währingerstr. 17, 1090 Vienna, Austria; 2 Vienna Doctoral School in Chemistry (DoSChem), University of Vienna, Währingerstr. 42, 1090 Vienna, Austria; 3 Department of Systems Biology, Harvard Medical School, Warren Alpert Building, 200 Longwood Ave, Boston, MA 02115, USA

**Keywords:** terahertz spectroscopy, induced dipoles, computational spectroscopy, water

## Abstract

We present a detailed examination of the absorption coefficients in the THz region for different water models using different types of potentials: the non-polarizable SPC/E, the Drude-polarizable SWM4-NDP and OPC3-pol, IPOL-0.13 and the multipole AMOEBA14 water. The primary focus is on understanding the interplay between permanent and induced dipole moments and their influence on the THz spectrum. Although the induced dipoles strongly contribute to the peak at 200 cm^−1^, merely increasing the induced dipole moments does not improve the agreement with experiments. We aim to investigate the behavior of the intensity at 200 cm^−1^ depending on the water model. Furthermore, we dissect the THz spectra of the water models into distinct contributions to gain more insight into the inter- and intramolecular interactions. Intermolecular interactions significantly contribute to the low-frequency peak, while the peak observed at 600 cm^−1^ can be adequately attributed to intramolecular dipole–dipole interactions.

## 1. Introduction

Terahertz (THz) spectroscopy, spanning frequencies between approximately 0.1 and 10 THz, lies between the mid-infrared and microwave regions of the electromagnetic spectrum and has garnered increasing scientific interest in recent years [1,2,3,4,5,6,7,8,9,10,11]. Due to the difficulty in generating and measuring radiation with the appropriate wavelength, the region between 0.3 and 10 (corresponding to 10 cm^−1^ to 330 cm^−1^) is often called the THz gap [12], where recording experimental spectra becomes challenging. As a result, fewer studies have focused on the application and interpretation of THz spectra compared to other frequency regimes [13], despite the critical insights this range offers into collective molecular relaxation phenomena, such as librational and translational motions [10,11,13]. The significance of the THz spectra of water in the range of 60 cm^−1^ to 300 cm^−1^ stems from the pivotal role played by its hydrogen bond network, which is responsible for most anomalous behaviors [14,15,16,17]. This frequency region provides insights into the collective intermolecular vibrations of the hydrogen bond network, in particular, where deviations from conventional bulk-like behaviors are anticipated [10,11,13,15,16,17,18]. Dynamic intermolecular collective polarization effects are responsible for the peak at approximately 200 cm^−1^. Ab initio molecular dynamics (AIMD) studies can, in most cases, reproduce these characteristics qualitatively [19,20,21], while classical force field-based molecular dynamics (MD) simulations often struggle [2,7,16,22,23]. More sophisticated water models, such as MB-pol [24], can reproduce the terahertz spectrum with reasonable accuracy. However, we chose to investigate simpler models to develop a proton exchange water model compatible with protex [25,26].

Librational motions primarily constitute the spectral range of 600 cm^−1^ to 900 cm^−1^, as demonstrated by experimental observations and classical MD simulations [7,17,19,27]. AIMD simulations appear to effectively model THz spectroscopy in this frequency domain, especially when significant vibrational correlations beyond the first solvation shell are not predominant [20]. For the SPC/E water model, it has been observed that water molecules forming three or four hydrogen bonds predominantly contribute to the peak at 700 cm^−1^ [7]. An increasing number of hydrogen bonds shifts the peak maximum to higher wave numbers. Inagaki et al. [17] assigned the Raman peak at 600 cm^−1^ to librational motions. The relaxation of collective polarizability is predominantly influenced by interactions between induced dipoles and permanent charges [17,20,28], leading to the significant autocorrelation of induced dipoles. The lower Raman peak observed at 200 cm^−1^ is mainly governed by interactions between induced dipoles [17]. This finding appears to be consistent with observations for the corresponding THz peak at the same frequency [28]. Table 1, adapted and expanded from Han et al. [29], presents different water models and their performances in the low-frequency region. As expected, more sophisticated models produce a more distinct translation peak at 200 cm^−1^ but comes with an increased computational cost. More information on the computation times can be found in the supporting material. To precisely identify the specific spectral contributions responsible for the observed differences, we performed a detailed decomposition of the spectra into all possible individual components. Our final goal is to develop a water model capable of proton exchange for use with protex, which also performs in the THz spectral range. Therefore, we investigated the interplay between permanent and induced dipole moments and their influence on the THz spectrum. Since protex requires a tetrahedral water model, we cannot adopt a bottom–up parameterization approach based on quantum mechanics. Instead, we must work within the constraints imposed by the tetrahedral geometry given by protex. Therefore, we chose water models closer or equal to the tetrahedral angle and focused on understanding how induced and permanent dipole moments influence the collective interactions within the hydrogen bond network.

In this study, we conduct a comprehensive examination of a non-polarizable and three polarizable water models for the computation of the absorption coefficient α(ω), which is intricately linked to the imaginary component of the permittivity $\epsilon^{\prime \prime }\left(\omega \right)$. Consequently, we adopted water models whose dielectric responses are in close agreement with experiments [7], thereby ensuring an accurate representation of collective dynamics. Among the candidates examined, the non-polarizable SPC/E and the polarizable SWM4-NDP models reproduce the dielectric spectrum with high fidelity, markedly outperforming the commonly employed TIP4P variants [7,51]. The static dielectric constants ϵ(0) of the widely utilized water models SPC/E and SWM4 are observed to be within the ranges of 72 to 74, and 78 to 79, respectively, [7,52]. These ranges are notably proximate to the experimental value of 78.3 [53], surpassing the fidelity of other prevalent models like TIP4P, TIP4P-2005, TIP5P, TIP5P-E, COS/G3, and SWM6 [7]. Additionally, the polarizable multipole AMOEBA14 [42] water model was included in this study, as it showed promising accuracy in the lower frequency region in recent studies [28]. SWM4 uses an H-O-H angle of 104.52°, corresponding to gas phase quantum mechanics calculations. At the same time, SPC/E has a tetrahedral angle of 109.47°, which we prefer for our future proton transfer water. While the molecular dipole is not significantly affected by this change in geometry, the collective dipole (and particularly its time correlation function) is affected. Although the angle of the multipole AMOEBA14 water model is 107.91°, Ponder et al. reported that its static dielectric constant aligns well with the experimental value as a result of their force field parameterization [42]. The absorption coefficient $\alpha \left(\omega \right)n^{\prime }\left(\omega \right)$ of AMOEBA14 water has previously been investigated by Esser et al. [54] and Sharma et al. [28] and was proven to sufficiently represent the terahertz spectrum. Multipole force fields are currently integrated only within a limited number of molecular dynamics packages: Amber [55], Tinker [56], and openMM [57]. Furthermore, we investigated two other Drude polarizable water models, the IPOL-0.13 [40] and the OPC3-pol [39] because they have an H-O-H angle of 109.47° and they are simpler models than SWM4-NDP with only three atoms, making them the most promising models to adapt to protex. A comparison of these water models with other existing models is presented in Table 1. The simulation details can be found in the Supplementary Materials.

## 2. Theory

### 2.1. The Absorption Coefficient α(ω)

Using plane wave propagation in an energy-absorbing medium [58], the absorption coefficient α(ω) as a function of the angular frequency ω

(1) $$\alpha \left(\omega \right)=\frac{2\omega }{c}\;n^{\prime \prime }\left(\omega \right)$$

can be obtained by the imaginary part $n^{\prime \prime }\left(\omega \right)$ of the refractive index $\hat{n}\left(\omega \right)=n^{\prime }\left(\omega \right)+i\;\cdot \;n^{\prime \prime }\left(\omega \right)$, which is linked to the complex permittivity $\hat{\epsilon }\left(\omega \right)=\epsilon^{\prime }\left(\omega \right)+i\;\cdot \;\epsilon^{\prime \prime }\left(\omega \right)$ [58]:

(2) $$\hat{\epsilon }\left(\omega \right)={\hat{n}}^{2}\left(\omega \right)=(n^{\prime }\left(\omega \right)+i\;\cdot \;n^{\prime \prime }\left(\omega \right){)}^{2}$$

Multiplying both sides of Equation (1) with $n^{\prime }\left(\omega \right)$ yields

(3) $$\alpha \left(\omega \right)\;\cdot \;n^{\prime }\left(\omega \right)=\frac{\omega }{c}\epsilon^{\prime \prime }\left(\omega \right)$$

and facilitates the computation of the absorption coefficient as the permittivity ϵ(ω) is a function of the auto-correlation function $\Phi_{D}\left(t\right)$ of the collective rotational dipole moment $M_{D}\left(t\right)$ [21,59]:

(4) $$\begin{array}{ccc} \Phi_{D}\left(t\right) & = & \langle {\vec{M}}_{D}\left(0\right)\;\cdot \;{\vec{M}}_{D}\left(t\right)\rangle \end{array}$$

(5) $$\begin{array}{ccc} \hat{\epsilon }\left(\omega \right)-\epsilon_{\infty } & = & \frac{1}{3Vk_{B}T\epsilon_{0}}\int_{0}^{\infty }-\frac{d\Phi_{D}\left(t\right)}{dt}\;e^{-i\omega t}\;dt \end{array}$$

(6) $$\begin{array}{ccc} & = & \frac{1}{3Vk_{B}T\epsilon_{0}}(\langle M_{D}^{2}\rangle +i\omega \int_{0}^{\infty }\Phi_{D}\left(t\right)\;e^{-i\omega t}\;dt) \end{array}$$

using the Laplace transform. The prefactor contains the Boltzmann constant $k_{B}$, the volume *V*, the temperature *T* of the system, and the dielectric permittivity of the vacuum $\epsilon_{0}$ = 8.85 × 10^−12^ AsV^−1^m^−1^. Combining Equations (3) and (6) results in

(7) $$\begin{array}{ccc} \alpha \left(\omega \right)\;\cdot \;n^{\prime }\left(\omega \right) & = & \frac{\omega^{2}}{3Vk_{B}Tc\;\epsilon_{0}}\int_{0}^{\infty }\Phi_{D}\left(t\right)\;\cos\left(\omega t\right)\;dt \end{array}$$

For convenience reasons, most publications use the Fourier transform [16,18,19,20,47,60,61] instead of the Laplace transform, resulting in a factor of two since the auto-correlation is an even function. Calculating the absorption coefficient α(ω) this way requires additional baseline corrections since the $\omega^{2}$ term in Equation (7) acts as a parabolic amplifier. A numerically more convenient approach exploits the properties of the Fourier transform and uses the time derivatives ${\vec{J}}_{D}\left(t\right)=d{\vec{M}}_{D}\left(t\right)/dt$ of the collective rotational dipole moments [18,60,61,62]

(8) $$\alpha \left(\omega \right)\;\cdot \;n^{\prime }\left(\omega \right)=\frac{1}{3Vk_{B}Tc\;\epsilon_{0}}\int_{0}^{\infty }\langle {\vec{J}}_{D}\left(0\right)\;\cdot \;{\vec{J}}_{D}\left(t\right)\rangle \cos\left(\omega t\right)\;dt$$

making the baseline correction unnecessary and is therefore preferred [60].

Typically, $\vec{J}\left(t\right)$ represents the electric current, which is not present in the simulations of aprotic water. Nevertheless, ${\vec{J}}_{D}\left(t\right)$ can be interpreted as the rotational current describing the rotation of the partial charges and, consequently the evolution of the dipole orientations over time and not their translation. In this way, ${\vec{J}}_{D}\left(t\right)$ bridges the gap between the molecular-level description of rotational dipolar motions and their manifestation in the macroscopic polarization of the sample. In contrast to the electric current $\vec{J}\left(t\right)$, the rotational current ${\vec{J}}_{D}\left(t\right)$ does not contribute to the frequency-dependent conductivity.

### 2.2. Decomposition of the Dipole Moment

**Figure 1:**
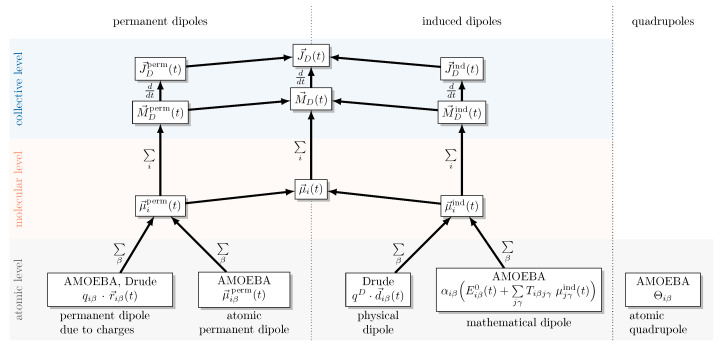
Decomposition of the collective rotational dipole moment ${\vec{M}}_{D}\left(t\right)$ into molecular dipole contributions indicated by the molecular index *i*. The permanent and induced components of the molecular dipole moments consist of atomic contributions (indicated by the index β), which depend on the polarizable force field type: AMOEBA or Drude.

As illustrated in Figure 1, the collective rotational dipole moment ${\vec{M}}_{D}\left(t\right)$ is obtained by summing the instantaneous molecular dipole moments ${\vec{\mu }}_{i}\left(t\right)$ of all molecules:

(9) $${\vec{M}}_{D}\left(t\right)=\sum_{i}{\vec{\mu }}_{i}\left(t\right)$$

Consequently, its time series cannot be averaged over the large ensemble of molecules, which increases the statistical noise in the dipole–dipole correlation function

$$\begin{array}{ccc} \langle \frac{{\vec{dM}}_{D}\left(0\right)}{dt}\;\cdot \;\frac{{\vec{dM}}_{D}\left(t\right)}{dt}\rangle & = & \langle {\vec{J}}_{D}\left(0\right)\;\cdot \;{\vec{J}}_{D}\left(t\right)\rangle = \end{array}$$

(10) $$\begin{array}{ccc} \langle \sum_{i}\frac{d{\vec{\mu }}_{i}\left(0\right)}{dt}\;\cdot \;\frac{d{\vec{\mu }}_{i}\left(t\right)}{dt} & + & \sum_{i}\sum_{j\neq i}\frac{d{\vec{\mu }}_{i}\left(0\right)}{dt}\;\cdot \;\frac{d{\vec{\mu }}_{j}\left(t\right)}{dt}\rangle \end{array}$$

(11) $$\begin{array}{ccc} & = & C^{\mathrm{self}}\left(t\right)+C^{\mathrm{cross}}\left(t\right) \end{array}$$

that underlies the simulated THz absorption spectrum and explains the limited availability of high-quality computational data in this frequency window. In particular, the cross-correlations $C^{\mathrm{cross}}\left(t\right)$ in the double sum are noisy at larger time intervals *t*.

Roke and co-workers [63] introduced correlated vibrational spectroscopy to disentangle self- and cross-correlations experimentally. They reported that the water cross-correlations level off beyond 1000 cm^−1^. Hence, the THz spectrum is transitioning to an IR spectrum, resulting in a diminution of the cross-correlations [60,61] above that threshold. Consequently, they can be neglected, and the auto-correlation of the molecular dipoles $C^{\mathrm{self}}\left(t\right)$ becomes a good approximation for the system’s response [60,61,62], allowing multiple time series or correlation functions to be averaged. However, a recent study on water emphasizes the importance of these cross-correlations

(12) $$\begin{array}{ccc} \alpha \left(\omega \right)\;\cdot \;n^{\prime }\left(\omega \right) & = & \frac{1}{3Vk_{B}Tc\;\epsilon_{0}}\left(\int_{0}^{\infty }C^{\mathrm{self}}\left(t\right)\;\cos\left(\omega t\right)\;dt+\int_{0}^{\infty }C^{\mathrm{cross}}\left(t\right)\;\cos\left(\omega t\right)\;dt\right)\\ & = & \alpha^{\mathrm{self}}\left(\omega \right)\;\cdot \;n^{\prime }\left(\omega \right)+\alpha^{\mathrm{cross}}\left(\omega \right)\;\cdot \;n^{\prime }\left(\omega \right) \end{array}$$

in the THz region [28], which we study later on.

An alternative partition distinguishes between permanent and induced components of the dipole moment. This would result in two auto-correlation and two cross-correlation functions. For the sake of simplicity, we correlate the total rotational dipole moment ${\vec{M}}_{D}\left(t\right)={\vec{M}}_{D}^{\mathrm{perm}}\left(t\right)+{\vec{M}}_{D}^{\mathrm{ind}}\left(t\right)$ with the permanent and induced dipolar contribution reducing the number of contributions

$$\begin{array}{cc} \; & \langle \frac{d{\vec{M}}_{D}\left(0\right)}{dt}\;\cdot \;\frac{d{\vec{M}}_{D}\left(t\right)}{dt}\rangle =\langle {\vec{J}}_{D}\left(0\right)\;\cdot \;{\vec{J}}_{D}\left(t\right)\rangle \end{array}$$

(13) $$\begin{array}{cc} = & \langle {\vec{J}}_{D}\left(0\right)\;\cdot \;{\vec{J}}_{D}^{\mathrm{perm}}\left(t\right)\rangle +\langle {\vec{J}}_{D}\left(0\right)\;\cdot \;{\vec{J}}_{D}^{\mathrm{ind}}\left(t\right)\rangle \end{array}$$

(14) $$\begin{array}{cc} = & \;C^{\mathrm{perm}}\left(t\right)+C^{\mathrm{ind}}\left(t\right) \end{array}$$

and yielding two spectral contributions $\alpha^{\mathrm{perm}}\left(\omega \right)\;\cdot \;n^{\prime }\left(\omega \right)$ and $\alpha^{\mathrm{ind}}\left(\omega \right)\;\cdot \;n^{\prime }\left(\omega \right)$. As experimental spectra in the THz region are typically described by the intensity depending on wavenumber $\bar{\nu }$ instead of the angular frequency ω, we will use $\bar{\nu }=\omega /\left(2\pi c\right)$ in the following investigations.

The molecular dipole moments (orange area in Figure 1) are commonly used to interpret the influence of the electrostatic properties of the molecules. However, the collective properties, which are gained by summation over the molecular index *i* should be used for the computation of the spectra. The actual dipolar forces in the polarizable MD simulations are computed on an atomic level (bottom area in Figure 1) depending on the type of polarizable force field: the harmonic Drude oscillator or the multipole AMOEBA model. The harmonic Drude oscillator model assigns to each atom iβ a partial charge $q_{i\beta }$ and an induced dipole ${\vec{\mu }}_{i\beta }^{\mathrm{ind}}\left(t\right)$ generated by the Drude oscillator pair. In contrast, AMOEBA employs a multipole description: every atom carries a permanent charge $q_{i\beta }$, a permanent dipole ${\vec{\mu }}_{i\beta }^{\mathrm{perm}}\left(t\right)$, and a quadrupole moment $\Theta_{i\beta }\left(t\right)$, while electronic polarization is treated through self-consistent induced dipoles ${\vec{\mu }}_{i\beta }^{\mathrm{ind}}\left(t\right)$.

### 2.3. Drude Polarizable Water Models

Most water models are parameterized from quantum mechanical calculations in the gas phase. However, water in its liquid form has properties different than water in the gas phase, since the H-bond network leads to increased O-H bond lengths as well as increased H-O-H angles. The first principles path integral MD simulations suggested that the O-H bond length is 1.014 Å and the H-O-H angle is 106.5° in liquid water [64]. Furthermore, we want to create a water model for protex which is going to have two dummy atoms, where proton exchange can take place. For this, we need a tetrahedral angle so that all hydrogen atoms in the molecule are equal. Therefore, we want to gain a deep understanding of how induced and permanent dipoles interplay and change the dynamics of the water.

In the Drude model, the molecular permanent dipole ${\vec{\mu }}_{i}^{\mathrm{perm}}\left(t\right)$ due to the partial charges is the sum of the product of the atomic partial charges $q_{i\beta }$ and the coordinates of the atom ${\vec{r}}_{i\beta }\left(t\right)$ in the non-charged molecule *i*:

(15) $${\vec{\mu }}_{i}^{\mathrm{perm}}\left(t\right)=\sum_{\beta }q_{i\beta }\;\cdot \;{\vec{r}}_{i\beta }\left(t\right)$$

The polarizable Drude water models discussed here use fixed geometries, i.e., the bond length O-H and the bond angle H-O-H are fixed. In this case, the molecular permanent dipole of water points in the direction of the bisector of the H-O-H angle.

**Figure 2:**
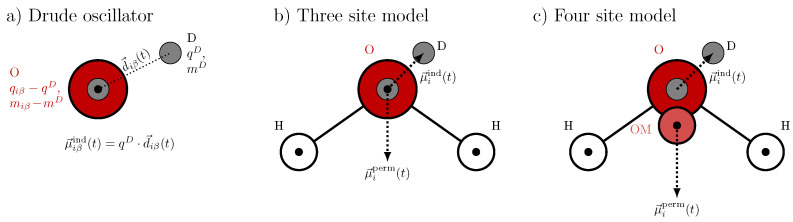
Polarizable Drude water models: (**a**) Drude oscillator pair (gray circles) attached to a polarizable oxygen (red). (**b**) Three-site polarizable water model (IPOL-0.13, OPC3-pol). (**c**) Four-site polarizable water model SWM4-NDP. The black circles indicate the position of the partial charges $q_{i\beta }$. Please note that O in SWM4-NDP has no partial charge but hosts the Drude pair.

The induced dipole moment is modeled by an oscillating Drude pair, as illustrated in Figure 2a. This pair consists of two oppositely charged particles: one Drude particle with charge $-q^{D}$, which is fixed at the position ${\vec{r}}_{i\beta }$ of the polarizable atom, and a second Drude particle with charge $q^{D}$ and mass $m^{D}$, which is harmonically bound to the first. The latter is free to oscillate about the former, mimicking the response of the electronic cloud to local electric fields. To conserve the total mass of the system, the mass of the polarizable atom is reduced by $m^{D}$ accordingly. Since typical Drude particle masses range from 0.2 to 0.4 amu, hydrogen atoms are commonly excluded from polarization treatments in Drude oscillator models due to their low mass. Consequently, in water, only the oxygen atom is modeled as polarizable. The corresponding induced dipole moment is given by

(16) $${\vec{\mu }}_{i\beta }^{\mathrm{ind}}\left(t\right)=q^{D}\;\cdot \;{\vec{d}}_{i\beta }\left(t\right)={\vec{\mu }}_{i}^{\mathrm{ind}}\left(t\right),$$

where ${\vec{d}}_{i\beta }\left(t\right)$ denotes the displacement vector between the two Drude particles at time *t*. Since this induced dipole arises from a pair of charges with equal magnitude and an opposite sign separated by a finite distance, it is referred to as a “physical dipole”.

We tested two three-site Drude polarizable water models with tetrahedral angles, namely the OPC3-pol [39] and the lesser known IPOL-0.13 [40] water model. The geometry is depicted in Figure 2b. They differ in the partial charges of the atoms and the polarizability of the oxygen. While the IPOL-0.13 model is quite similar to other Drude polarizable water models with low Drude masses $m^{D}$, OPC3-pol uses half of the mass of oxygen, which led to slightly less stable simulations. Furthermore, the separation between the induced and permanent dipole is not straightforward when using this partition. In addition, we also tested the SWM-NDP model, which is a four site model. As illustrated in Figure 2c, the additional artificial site OM is 0.24 Å moved towards the hydrogen atoms and carries the charge of the oxygen as tabulated in Table 2. To reproduce the permanent dipole ${\vec{\mu }}_{i}^{\mathrm{perm}}$ of water, this charge and the charge of the hydrogen atoms are increased due to the shorter distances between the positive and negative charge centers. Nevertheless, the Drude oscillator pair is attached to the original oxygen atom O. Consequently, induced and permanent dipoles in SWM-NDP may be slightly more decoupled compared to three site models.

### 2.4. Amoeba Water Models Using Multipoles

The AMOEBA (Atomic Multipole Optimized Energetics for Biomolecular Applications) force field [42] represents an advanced formalism for calculating the potential energy of a system of molecules. It accurately models electrostatic interactions using distributed multipole expansions [65,66] rather than relying solely on point charges, enabling the AMOEBA force field to capture the anisotropy of electrostatic interactions more precisely. Multipole moments are mathematical constructs that describe the spatial distribution of charge within a physical system [67,68]:

(17) $$\begin{array}{ccc} {\vec{\mu }}_{i}\left(t\right) & = & {\vec{\mu }}_{i}^{\mathrm{perm}}\left(t\right)+\sum_{\beta }{\vec{\mu }}_{i\beta }^{\mathrm{perm}}\left(t\right)+\sum_{\beta }{\vec{\mu }}_{i\beta }^{\mathrm{ind}}\left(t\right) \end{array}$$

(18) $$\begin{array}{ccc} & = & \sum_{\beta }q_{i\beta }\;\cdot \;{\vec{r}}_{i\beta }\left(t\right)+\sum_{\beta }{\vec{\mu }}_{i\beta }^{\mathrm{perm}}\left(t\right)+\sum_{\beta }{\vec{\mu }}_{i\beta }^{\mathrm{ind}}\left(t\right) \end{array}$$

**Figure 3:**
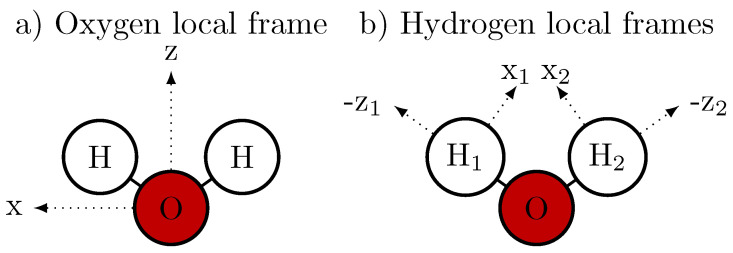
Local frames for the AMOEBA [66,69] atomic dipoles ${\vec{\mu }}_{i\beta }$: (**a**) The *z* axis for the local oxygen frame is the bisector of the H-O-H angle. The *x* axis is in the plane of the atoms and perpendicular to the *z* axis, which is not necessarily in the same direction as the HH vector (e.g., two different bond lengths). (**b**) The *z* axis is always in the direction from the respective hydrogen to the oxygen. The *x* axis is in the plane of the atoms and pointing inwards. The *y* axis for oxygen and H1 points up, and for H2 points down.

The atomic monopole moment represents the partial charge of the atom $q_{i\beta }$. Their spatial distribution ${\vec{r}}_{i\beta }\left(t\right)$ results in a permanent molecular dipole moment ${\vec{\mu }}_{i}^{\mathrm{perm}}\left(t\right)$. The atomic dipole moment ${\vec{\mu }}_{i\beta }^{\mathrm{perm}}\left(t\right)$ provides an additional vectorial description of the electron density at that atom and is the first multipole moment to include directionality in the electrostatic field [62]. Please note that the magnitudes of the atomic ${\vec{\mu }}_{i\beta }^{\mathrm{perm}}$ are fixed, but they rotate with the water molecule *i* and are thus time dependent. The orientation of the atomic dipole moment ${\vec{\mu }}_{i\beta }^{\mathrm{perm}}$ in the local atomic frame (which is tabulated in Table 2) is shown in Figure 3.

In contrast to the Drude oscillator model, the induced dipoles ${\vec{\mu }}_{i\beta }^{\mathrm{ind}}\left(t\right)$ are realized by mathematical dipoles, i.e., a vector and not a pair of oppositely charged particles. It can be computed in a self-consistent manner:

(19) $$\begin{array}{ccc} {\vec{\mu }}_{i\beta }^{\mathrm{ind}}\left(t\right) & = & \alpha_{i\beta }\left({\vec{E}}_{i\beta }^{0}\left(t\right)+{\vec{E}}_{i\beta }^{ind}\left(t\right)\right) \end{array}$$

(20) $$\begin{array}{ccc} \; & = & \alpha_{i\beta }({\vec{E}}_{i\beta }^{0}\left(t\right)+\sum_{j\gamma \neq i\beta }^{N}\overset{↔}{T}({\vec{r}}_{i\beta j\gamma }\left(t\right))\;{\vec{\mu }}_{j\gamma }^{\mathrm{ind}}\left(t\right)) \end{array}$$

Here, $\alpha_{i\beta }$ is the atomic polarizability, ${\vec{E}}_{i\beta }^{0}\left(t\right)$ is the electric field arising from the permanent partial charges, and ${\vec{E}}_{i\beta }^{ind}\left(t\right)$ represents the local electric field induced by the other induced dipoles. $\overset{↔}{T}\left({\vec{r}}_{i\beta j\gamma }\left(t\right)\right)$ is the dipole–dipole tensor formed by the dyadic product:

(21) $$\overset{↔}{T}\left({\vec{r}}_{i\beta j\gamma }\left(t\right)\right)=\frac{1}{r_{i\beta j\gamma }^{3}\left(t\right)}(3\frac{{\vec{r}}_{i\beta j\gamma }\left(t\right)\;\cdot \;({\vec{r}}_{i\beta j\gamma }\left(t\right){)}^{T}}{r_{i\beta j\gamma }{\left(t\right)}^{2}}-\overset{↔}{I})$$

where ${\vec{r}}_{i\beta j\gamma }\left(t\right)$ is the vector joining atoms iβ and jγ.

The AMOEBA water model employs multipoles up to quadrupole moments (see Figure 1). The quadrupole moment $\Theta_{i\beta }$ offers a more refined representation by capturing how the electron density is distributed in a quadrilateral pattern. This enables the modeling of more complex charge distributions that partial charges and atomic dipoles cannot capture. The sum of the diagonal elements of $\Theta_{i\beta }$ is zero by definition [65]. Higher multipole moments are usually not implemented in MD simulations.

Several AMOEBA water models exist [42,66,68,69,70,71]: The AMOEBA water model was first parameterized by Pengyu and Ponder [69] and the parameters were later forced-balanced [42] to improve certain physical quantities. Other improvements on the water model include introducing anisotropic atomic polarizabilities [71], and Gaussian distributed multipoles [70]. Furthermore, in the inexpensive water model iAMOEBA [43], the induced dipoles are determined directly from the permanent multipole electric fields and do not interact with one another. However, the increased accuracy of the AMOEBA force field comes at the cost of greater computational complexity and resource requirements compared to simpler force fields based on point charges. As the computational power increases, the AMOEBA force field and other multipole-based methods are becoming more accessible. In this study, we focused on the AMOEBA14 [42] water model as it has been shown to describe the THz spectrum accurately [28,54]. We implemented the AMOEBA14 force field in openMM [57], therefore we used 1/3 of the values of the quadrupole tensor from Table 2.

We also analyzed the trajectories from the non-polarizable SPC/E model consisting of three atoms. Since induced dipoles do not exist during trajectory production in this case, the corresponding correlation function was calculated from induced dipole moments post-trajectory production according to Ref. [17], which uses the self-consistent field method of the AMOEBA-induced dipoles in Equation (19). This method may also be applied to machine learning trajectories to obtain induced dipoles.

## 3. Results and Discussion

### 3.1. The Marginal Impact of Charge Transfer

The first studies on the charge transfer of water have been reported by Rick and co-workers using a fluctuating charge model [72,73,74], which was also used to study the charging at the surface of pure water [75] and the stabilization of weakly bound complexes of water [76]. However, Torii [18,48] reported that the characteristic 200 cm^−1^ peak could already be reproduced using the non-polarizable TIP4P water model in conjunction with a post-trajectory charge transfer algorithm. The algorithm is based on the spatial proximity between the hydrogen bond-accepting oxygen atom and the hydrogen atoms of neighboring water molecules. The estimated charge transfer associated with each hydrogen bond is approximately Δq≈ 0.01 e, which induces a corresponding change in the water dipole moment of about 0.14 D. Furthermore, the polarizable TL4P^i^-CT model [37] explicitly accounts for intermolecular charge transfer effects.

**Figure 4:**
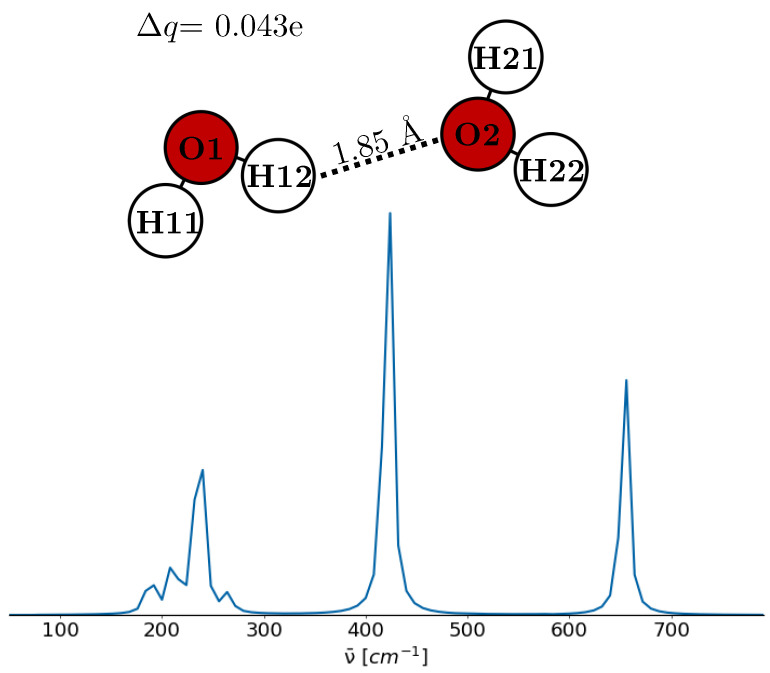
Simple model of a water dimer and its predicted THz spectrum. Partial charges and dipole moments are given in Table 2.

To elucidate the significance of the charge transfer, we employed a simplified model of a water dimer, as shown in Figure 4. The dimer’s geometry and THz spectrum were determined through quantum-chemical optimization and frequency calculations using B3LYP/6-311g(d,p) with a universal solvation model of water [77]. Partial charges were determined by the CHELPG method and are similar to those of the SPC/E water in Table 2. CHELPG charges were used because they provide the most accurate partial charges for isolated water molecules and water dimers [78,79]. The derived geometry aligns with the linear non-planar geometry identified as a global minimum by Ghosh et al. [80]. Applying the Torii algorithm to this geometry, a charge transfer Δq of 0.036 e was predicted, comparable to the 0.043 e deduced from CHELPG charges. However, the variations in water dipole moments were less pronounced than anticipated, approximately 0.02 D. It is important to note that only a single hydrogen bond was analyzed in this simple model. The THz spectrum depicted in Figure 4 reveals a spectral profile closely resembling that of liquid-phase water. A detailed analysis of this spectrum identifies distinct vibrational modes corresponding to various peaks. Specifically, the peak at 188 cm^−1^ stems from an out-of-plane motion of H11 combined with a rocking motion in the second water molecule. The peak at 211 cm^−1^ is characterized by an oxygen–oxygen vibrational mode. Furthermore, at 236 cm^−1^, the observed normal mode encompasses a rocking motion of the first water molecule and a wagging motion in the second. The peak at 265 cm^−1^ combines a rocking motion in the first water molecule and a twisting motion in the second. The prominent peak at 422 cm^−1^ has almost the same characteristics observed at 236 cm^−1^. Lastly, at 654 cm^−1^, the vibrational activity is marked by H12 oscillating along the oxygen–oxygen axis, while the second water molecule exhibits a twisting motion. The reasonable concordance between the THz spectrum of our simplified model and the experimental spectrum suggests that the modeled geometry and charges reflect experimental conditions. Consequently, the small change between the molecular dipoles implies that charge transfer may not be the dominant mechanism to enforce the THz peak at 200 cm^−1^. The ability of the polarizable, charge-transfer-capable TL4P^i^-CT model [37] to reproduce this peak may be attributed to the contribution of induced dipole moments arising from the model’s explicit treatment of polarization effects.

Roke and co-workers [63] investigated the influence of ionic species on the vibrational response. The addition of a 2 M aqueous NaOH solution induced a blueshift of approximately 15 cm^−1^ in the 200 cm^−1^ peak, whereas the introduction of 2 M aqueous HCl caused a redshift of about 10 cm^−1^. These spectral shifts are attributed not to charge transfer but rather to the effects of complete proton transfer and the specific Coulomb interactions with hydrated protons (H^+^)_aq_ or hydroxide ions (OH^−^)_aq_.

### 3.2. The Importance of Polarization

**Figure 5:**
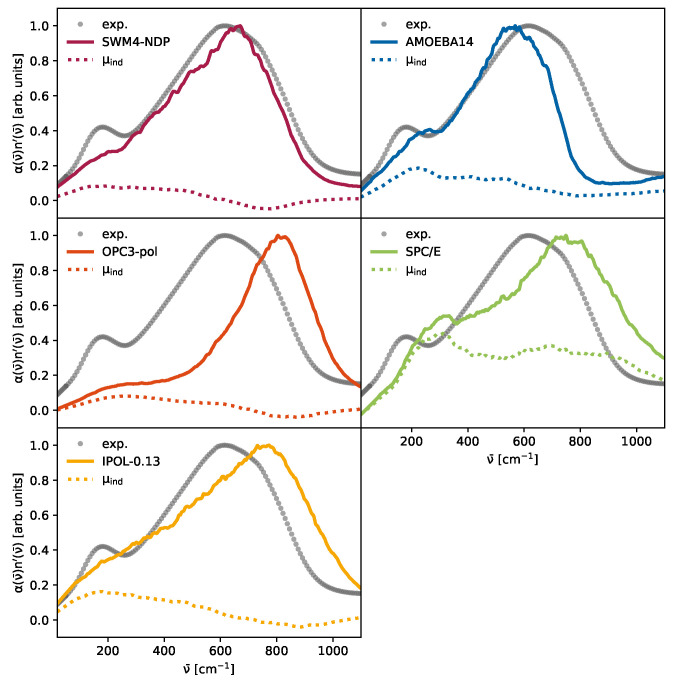
Scaled absorption coefficient of the water models: The solid lines represent $\alpha \;\cdot \;n^{\prime }$ for the respective water model. The dotted lines are the contributions of the induced dipole interactions. The gray dots are experimental values and were taken from Bertie and Lan [82].

The peak at approximately 200 cm^−1^ has previously been associated with intermolecular hydrogen-bond stretching vibrations [8,9,11,13], as well as delocalized collective dynamics [10,11,13,23], as confirmed by dielectric [11], two-dimensional IR and optical Kerr effect spectroscopy [8,11,13]. The observed spectral features in this region are strongly influenced by dipolar coupling and fast spectral diffusion processes. Our simulations corroborate these findings, showing that only models incorporating induced dipoles capture the intensity and position of this peak with reasonable fidelity, highlighting the role of intermolecular polarization dynamics. The congruence between the computational $\alpha \;\cdot \;n^{\prime }$ obtained from the auto-correlation function of the collective rotational dipole moment according to Equation (8) and the experimental data (gray dotted line) is presented in Figure 5. The computational spectra are smoothed by a Savitzky–Golay filter to reduce the noise. The spectra are scaled by their maxima for better comparison. The decomposed spectra with unscaled intensities are in the Supplementary Materials (Figures S1, S3, S5, S7, S9, S11 and S13). The three spectra on the left hand side of Figure 5 are from Drude-polarizable force fields. All spectra display only a shoulder in the total spectrum, rather than the distinct dual peak structure that is expected. Additionally, the contribution from induced dipole moments is lower compared to the other two spectra. From these models, SWM4-NDP (red) performs the best at 600 cm^−1^ to 900 cm^−1^; however, the peak at 200 cm^−1^ is severely underestimated and is merely a shoulder. The OPC3-pol (orange) water model underestimates the relative intensity of the peak at 200 cm^−1^ and the libration peak is blue-shifted. While the relative intensity of the IPOL-0.13 (yellow) model at 200 cm^−1^ is quite close to the experimental value, there is no visible peak separation. For the non-polarizable SPC/E model (green), a post-simulation computation of induced dipoles was performed, applying Equation (20). These induced dipoles are essential in the emergence of a pronounced peak at approximately 260 cm^−1^, which overestimates the intensity of the experimental peak observed at 200 cm^−1^. This may be due to the elevated polarizability $\alpha_{i}$ of 1.14 ${Å}^{3}$, which exceeds the scaled polarizability of the SWM4-NDP water model of 0.98 ${Å}^{3}$; however, the polarizability of AMOEBA14 (1.47 ${Å}^{3}$) is higher than the one chosen for the post-production polarized SPC/E and the relative intensity of the AMOEBA14 water model is lower than that of SPC/E. During the parameterization of the DGenFF force field, the quantum-mechanically derived polarizabilities are scaled down by a factor of 0.85 to enhance the agreement with the liquid phase [81]. The second peak around 650 cm^−1^ in SPC/E water already emerged from the pure non-polarizable trajectory [7], but is still visible in our “polarizable” SPC/E model. Although no water model perfectly describes the THz spectrum, considering both intensity and frequency, the AMOEBA14 water model in Figure 5 (blue) captures the dual-peak shape. The intensities of both peaks are very close to experimental data; however, the frequencies are slightly shifted, the first peak is blue-shifted, while the second peak already levels off at 800 cm^−1^. As the multipole model does not incorporate intermolecular charge fluxes, the assertions made by Torii [18] and Car and co-workers [19,27] on the importance of charge transfer could not be confirmed. We propose instead that the induced contributions are the primary source of the peak observed at 200 cm^−1^. In this region, the induced dipole contributions of all water models in Figure 5 exhibit a peak maximum.

### 3.3. The Competition Between Induced and Permanent Dipoles

**Figure 6:**
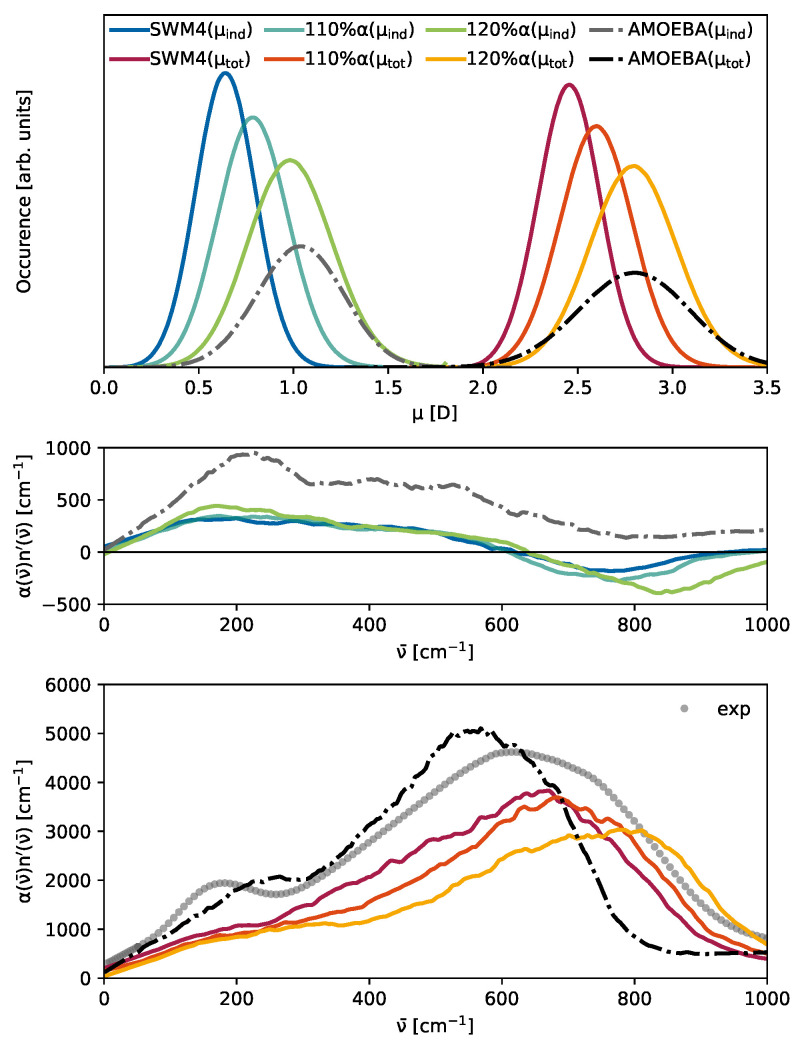
(**Top**): Histograms of the total (SWM4: red, orange, yellow with increasing polarizability, AMOEBA: black) and induced dipole moments (SWM4: blue, turquoise, green with increasing polarizability, AMOEBA: gray). (**Middle**): THz contribution of the induced dipoles $\alpha^{\mathrm{ind}}\;\cdot \;n^{\prime }$ of SWM4-NDP and AMOEBA with the same color code as above. (**Bottom**): The total THz spectrum $\alpha \;\cdot \;n^{\prime }$ of the polarizable SWM4-NDP and multipole AMOEBA14. The gray dots depict the experimental data.

**Figure 7:**
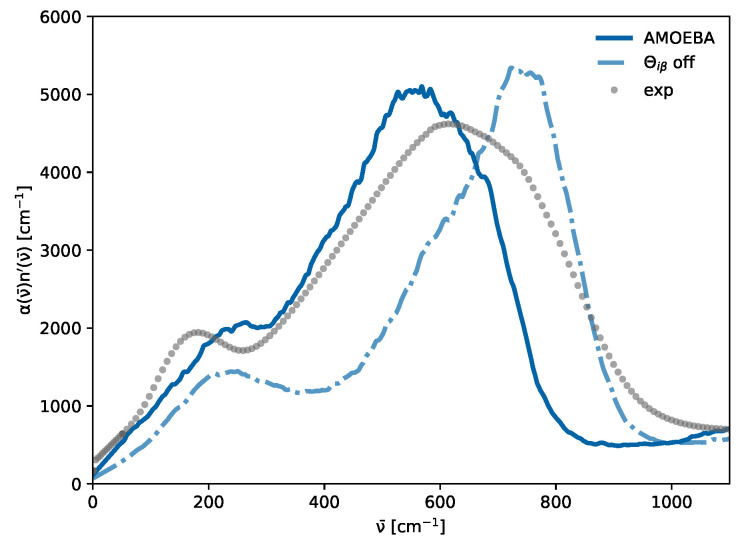
Absorption spectrum of the multipole AMOEBA water model with and without atomic quadrupoles $\Theta_{i\beta }$.

While not immediately apparent for the total THz spectrum in Figure 5, all water models exhibit a peak at 200 cm^−1^ upon restriction to the contribution of induced dipoles (dotted lines). A plausible hypothesis for the hidden peak at 200 cm^−1^ observed in Figure 5 for the polarizable SWM4 model might be attributed to the relatively weak molecular induced dipoles ${\vec{\mu }}_{i}^{\mathrm{ind}}\left(t\right)$ in comparison to the permanent dipoles ${\vec{\mu }}_{i}^{\mathrm{perm}}\left(t\right)$, which can be seen in Figure 6 in the top panel. The intensities in the THz spectrum emerging from induced dipoles of the SWM4 model (blue line) are only half as strong as those of the AMOEBA model (gray dash-dotted line). This is also reflected in the total dipole moments. In addition, as visible in Table 2, the permanent dipole of the polarizable SWM4 model is significantly lower than the non-polarizable SPC/E model, which the induced dipoles within the SWM4 model should compensate for. To analyze the effect of the induced dipoles, we increased the polarizability within the SWM4 model with increments of 10% (orange line) and 20% (yellow line), as depicted in Figure 6. In the top panel, the green line represents the induced dipole moment distribution of SWM4-NDP scaled by 120% and the yellow line represents the total dipole moment distribution. At this scale, they are similar to the distributions of the AMOEBA14 water model (gray and black dash dotted lines). Contrary to expectations, this polarizability increase did not result in a corresponding elevation of the relative height of the peak at 200 cm^−1^, but an unintended shift in the librational peak at 800 cm^−1^ to higher wavenumbers was observed instead. We want to emphasize that, by changing the polarizability, other physical properties such as permittivity and relaxation times were also affected. This outcome elucidates that, while induced dipoles play an essential role in forming the 200 cm^−1^, simply enhancing the strength of these induced dipoles does not directly translate to an increased prominence of this peak. Consequently, this suggests that a more nuanced approach, potentially involving the implementation of multipole interactions, may be required to achieve a significant amplification of the 200 cm^−1^ peak in the spectral profile. This insight underscores the complexity of molecular interactions within the model and points towards the need for advanced modeling techniques to accurately capture the spectral characteristics of water. In the multipole AMOEBA model, not only the partial charges but also the atomic dipole moments contribute to the permanent molecular dipole moments ${\vec{\mu }}_{i}^{\mathrm{perm}}\left(t\right)$. This allows for a more flexible generation of these dipole moments, as neither additional particles (as, for example, the OM dummy atom in SWM4, as can be seen in Figure 2c) nor extreme partial charges have to be employed. The interaction of the induced dipoles with the partial charges and the permanent atomic dipoles differs not only in strength but also in their distance dependence. In addition to the atomic dipoles, the AMOEBA water model also features quadrupoles (see Table 2), which have a significant impact on the THz spectrum, as shown in Figure 7. Turning all $\Theta_{i\beta }$ off results in shifts in the two peaks. However, the low-frequency peak is only marginally affected, whereas the librational peak redshifts by approximately 200 cm^−1^. This leads to less overlap between the two peaks. The 200 cm^−1^ peak is only a shoulder in the original multipole AMOEBA water model and becomes more apparent when no quadrupoles are used. One may fine-tune the quadrupoles’ strengths to improve the AMOEBA model’s agreement with the experiment. However, this way, one may sacrifice the agreement of the original model with other experimental data, e.g., diffusion coefficients. The AMOEBA multipole model offers a distinct advantage because of its quadrupole moments comprise individual atomic contributions. This approach allows for more precise parameterization to capture the (anisotropic) electronic response of the molecule. In contrast, the other models incorporate molecular quadrupole moments derived from the partial charge distribution at specific positions. These models lack the flexibility to adjust quadrupole moments at the level of atomic contributions. Nevertheless, as illustrated in Figure 7, the atomic quadrupole moments substantially shape the THz spectrum.

### 3.4. Cross- and Self-Terms

Evaluating the cross-correlation functions between the dipole moments of different molecules is essential for accurately representing the THz spectrum of water, particularly the peak at 200 cm^−1^, as highlighted by Sharma et al. [28]. While individual molecular dipole moments can often adequately describe IR spectra, collective molecular motions primarily influence the THz region.

**Figure 8:**
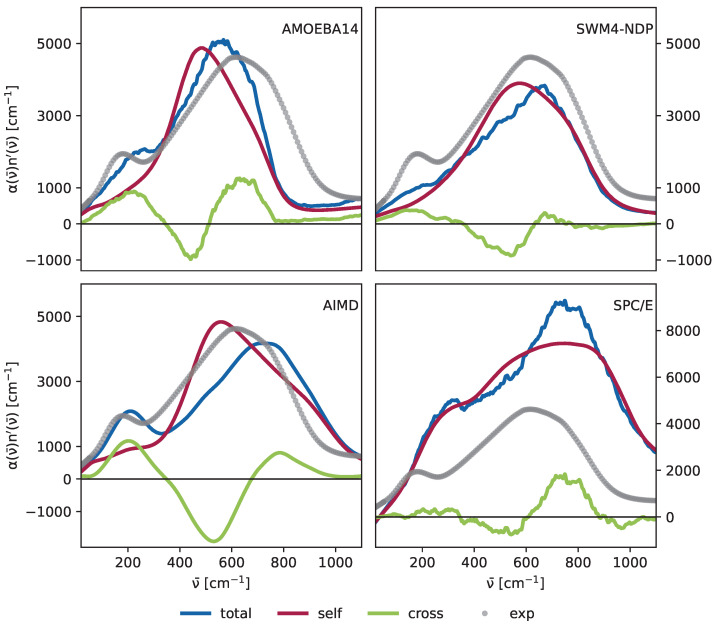
(**Top left**): AMOEBA14, (**top right**): SWM4-NDP, (**bottom left**): AIMD, (**bottom right**): SPC/E. The green lines represent the cross-terms, and the red lines represent the self-terms. The blue lines are the sum of the two contributions and yield the total spectra. The gray dots depict the experimental data from Bertie and Lan [82]. The data from AIMD simulations were kindly provided by Shane Carlson and Roland Netz and are published in Carlson et al. [21].

In Figure 8, we compare the spectra of the AMOEBA14, SWM4-NDP, and SPC/E force fields to AIMD simulations. The total spectrum (blue) is decomposed into contributions from self-terms (dark red) and cross-terms (green), as defined in Equation (12). The cross-terms are significantly more pronounced in the AMOEBA14 spectrum compared to the other models, resulting in a distinct peak at 200 cm^−1^, unlike the SWM4-NDP spectrum, where only a minor shoulder is observed at this frequency. Although the “polarizable” SPC/E water shows a peak between 200 cm^−1^ to 400 cm^−1^, the cross correlations do not contribute in this force field. This is possibly due to the post production added polarizability. The detailed spectra are in the Supplementary Materials (Figures S1, S3, S5, S7, S9, S11 and S13), where it is clear that only SPC/E water has a significant contribution to the peak at 200 cm^−1^ caused by induced self-terms, while in all other models, it arises from the induced cross-terms. AIMD calculations from Carlson et al. [21] support that cross-terms mainly shape the peak at the low-frequency region. While there is a contribution from self-terms at 200 cm^−1^, the shape in this frequency range is rather flat, therefore the distinctive peaks arise from cross-terms. Another important reason for the distinct dual-peak is the following negative contribution at 400 cm^−1^ to 600 cm^−1^ as it leads to better peak separation. While all models we tested show this behavior in a way, the cross term contribution is most pronounced in the AMOEBA14 model. The libration peak at 600 cm^−1^ predominantly arises from the self-terms of the correlation functions. Nevertheless, there are cross-term contributions in this region as well. The contributions of the AMOEBA14 model are stronger in that region compared to the SWM4-NDP model, and especially the negative contribution at 500 cm^−1^ is important for the peak separation. Interestingly, Roke and co-worker [63] reported the experimental evidence of the double peak of cross-correlations below 1000 cm^−1^, which is best reproduced by the AMOEBA multipole force field and the AIMD study.

The dominance of the self-terms at 600 cm^−1^ reinforces its attribution to the rotational motion of individual water molecules. Conversely, the peak at 200 cm^−1^ is linked to network interactions, underscoring the necessity of evaluating collective motions to capture this feature accurately.

### 3.5. Dipole Moments

The average dipole moments calculated from the dipole moments of the simulation from the arithmetic mean are listed in Table 3. The dipole moments of the water models are not constant during the simulation, but rather show a distribution. The detailed distributions for each water model can be found in the Supplementary Materials (Figures S2, S4, S6, S8, S10, S12 and S14). For rigid water models, the permanent dipoles are constant, but the induced dipole moments change during the simulation. AMOEBA14, which is the only non-rigid water model we studied, has a fluctuating permanent dipole moment due to bending and stretching of the molecule. Therefore, there is a dipole distribution for permanent dipole moments as well, leading to different results from the model values described in Table 2. Given that AMOEBA14 outperformed the other water models, we evaluated the performance of an unconstrained version of the SWM4-NDP model for comparison, as well as a constrained AMOEBA14 version. The difference in the resulting THz spectra was negligible. A comparison between the constrained and unconstrained SWM4-NDP and AMOEBA14 spectra is provided in the Supplementary Materials (Figure S15). While the absolute values of the dipole moments do influence the shape of the spectrum, there is no obvious relation between the value of the dipole moments and the intensities of the two peaks. As already mentioned and depicted in Figure 6, increasing the induced dipole moment itself does not necessarily lead to higher intensities at 200 cm^−1^.

## 4. Conclusions

We presented a comprehensive and methodologically refined analysis of the THz absorption spectrum of various water models, with particular emphasis on the interplay between permanent and induced dipole moments and the role of their self- and cross-correlation functions. While the conventional MD simulation using non-polarizable and polarizable force fields provides valuable insights into the THz absorption spectra of water, several limitations must be acknowledged. Non-polarizable models such as SPC/E, though computationally efficient and successful in reproducing certain static properties (e.g., dielectric constants), inherently lack the ability to describe dynamic polarization and charge redistribution effects. As a result, they fail to capture the subtle but critical features of the low-frequency spectrum, particularly the peak near 200 cm^−1^, unless polarization is artificially added post hoc. The disadvantage of adding polarization later on is that the intensities of the spectra are not comparable anymore and therefore only relative spectra can lead to comparable results.

Polarizable models, especially those employing Drude oscillators (e.g., SWM4-NDP, OPC3-pol, IPOL-0.13), offer improved accuracy in capturing induced dipole contributions. However, our analysis reveals that increasing polarizability alone does not straightforwardly enhance spectral agreement. In fact, it can cause undesired shifts in librational peaks. This indicates that many-body effects and the balance between permanent and induced components must be tuned in a nontrivial manner.

Among the models studied, only the multipolar AMOEBA14 force field achieves a level of accuracy in modeling the collective electrostatic behavior of water comparable to AIMD simulations. The use of distributed multipoles in AMOEBA14 enables flexible and targeted parameterization to reproduce both spectral and dynamical properties. Although AMOEBA14 does not account for intermolecular charge transfer, this limitation does not significantly impact its performance in the THz frequency range. However, interactions with solvated protons or hydroxide ions, accessible in AIMD but absent in AMOEBA14, remain outside its current scope. To address this limitation, we propose the development of a new, proton-exchange-capable water model within the protex framework, based on the multipolar AMOEBA force field.

Overall, our findings provide new insights into the microscopic origin of low-frequency spectroscopic features of water and offer a clear pathway towards the design of advanced, polarizable water models. These results establish a firm theoretical basis for extending multipole-based force fields to accurately describe proton-coupled systems, thereby representing a substantial advancement in computational spectroscopy and the development of next-generation force field models.

## Figures and Tables

**Table 1:** Reprinted (adapted) with permission from Han et al. [29]. Copyright 2023 American Chemical Society. ^*a*^ For non-rigid models: the equilibrium angle of the water model as a monomer. ^*b*^ Liquid phase angle. ^*c*^ Ground state vibrationally averaged geometry. ^*d*^ Based on water hexamers. Appearance of the peak at 200 cm^−1^ for different water models. For the AMOEBA14 water model, the H-O-H angle is flexible, however, there is an equilibrium angle. As the MB-pol water model is not calculated from a classic harmonic potential, there is no universal equilibrium angle.

Water Model	Reference	H-O-H Angle ^*a*^ [deg]	200 cm^−1^
**Rigid, non-polarizable**			
SPC/E	[29]	109.47 [30]	no
TIP4P/2005	[29]	104.52 [31]	no
**Flexible, non-polarizable**			
SPC/Fw	[32]	113.24 [32]	no
TIP4P/2005f	[29]	107.4 [33]	no
**Rigid, with post-MD induced dipoles**			
SPC/E-$\mu^{ind}$	this work	109.47	yes
TIP4P/2005-$\mu^{ind}$	[34]	104.52	yes
E3B-$\mu^{ind}$	[35]	104.52	shoulder
**Rigid, polarizable**			
SWM4-NDP	This work, [34]	104.52 [36]	shoulder
SWM4-NDP^+10%*pol*.^	This work	104.52	shoulder
SWM4-NDP^+20%*pol*.^	This work	104.52	yes
TL4P	[37]	105.3 ^*b*^ [38]	shoulder
OPC3-pol	This work	109.47 [39]	shoulder
IPOL-0.13	This work	109.47 [40]	shoulder
**Flexible, polarizable**			
AMOEBA14	This work, [9,23,28,41]	107.91 [42]	yes
AMOEBA14 (Θ off)	This work	107.91	yes
iAMOEBA	[43]	106.48 [43]	yes
AMOEBA+	[41]	108.81 [44]	yes
**Flexible, polarizable with charge flux**			
POLI2VS	[45]	104.52 [45]	yes
AMOEBA+CF	[41]	104.54 [41]	shoulder
TTM3-F	[46]	104.5078 [47]	shoulder
**Rigid, polarizable with charge transfer**			
TL4P^i^-CT	[37]	105.3 ^*b*^ [38]	yes
TIP4P-$\mu^{ind}$/CT	[48]	104.52	yes
**Explicit, many-body potential**			
MB-pol	[49]	104.69 ^*c*^ [24]	yes
WHBB	[50]	- ^*d*^	yes

**Table 2:** Partial charges and dipoles of the MD water models. The atomic dipoles and quadrupoles use their individual atomic local frames (see Figure 3).

Atom Type	$q_{i\beta }$	$|{\vec{\mu }}_{i\beta }|$	$\Theta_{i\beta }$	$|{\vec{\mu }}_{i}^{\mathrm{perm}}|$
	[e]	[D]	[D Å]	[D]
**SPC/E [30]**				
OT	−0.848			2.36
HT	0.424			
**SWM4 [36]**				
O	0.00000			
OM	−1.11466			1.85
H	0.55733			
**IPOL-0.13 [40]**				
O	−0.669			1.86
H	0.3345			
**OPC3-pol [39]**				
O	−0.610			2.05
H	0.305			
**AMOEBA14 [42]**				
O	−0.42616		xx = 0.236	1.71
		*z* = 0.159	yy = −0.312	
			zz = 0.075	
H	0.21308	*x* = −0.257	xx = 0.165	
			yy = 0.120	
		*z* = −0.691	xz = −0.094	
			zz = −0.286	
**Water dimer**				
O1	−0.810			
H11	0.384			2.37
H12	0.383			
O2	−0.752			
H21	0.394			2.35
H22	0.401			

**Table 3:** The absolute values of permanent, induced, and total dipole moments of all water models. For rigid water models (SPC/E, SWM4-NDP all variants, OPC3-pol, IPOL-0.13), the permanent dipole moment is constant, whereas for nonrigid water models (AMOEBA) there is a dipole distribution. The values in the table are the mean values.

Model	$\left|{\vec{\mu }}_{i}^{\mathrm{perm}}\right|$ [D]	$\left|{\vec{\mu }}_{i}^{\mathrm{ind}}\right|$ [D]	$\left|{\vec{\mu }}_{i}\right|$ [D]
SPC/E (added polarizability)	2.35	0.79	3.15
SWM4-NDP	1.85	0.65	2.50
SWM4-NDP^+10%*pol*.^	1.85	0.79	2.60
SWM4-NDP^+20%*pol*.^	1.85	0.99	2.80
OPC3-pol	2.05	1.09	3.11
IPOL-0.13	1.86	1.22	3.05
AMOEBA14	1.80	1.04	2.79

## Data Availability

The data that support the findings of this study are available from the corresponding author upon reasonable request.

## Supplementary Materials

The following supporting information can be downloaded at: https://www.mdpi.com/article/10.3390/molecules30142941/s1, Figure S1: Individual contributions to the THz spectrum of SPC/E water; Figure S2: Dipole histograms of SPC/E water; Figure S3: Individual contributions to the THz spectrum of SWM4-NDP water; Figure S4: Dipole histograms of SWM4-NDP water; Figure S5: Individual contributions to the THz spectrum of SWM4-NDP water with 10% increased polarizability; Figure S6: Dipole histograms of SWM4-NDP water with 10% increased polarizability; Figure S7: Individual contributions to the THz spectrum of SWM4-NDP water with 20% increased polarizability; Figure S8: Dipole histograms of SWM4-NDP water with 20% increased polarizability; Figure S9: Individual contributions to the THz spectrum of AMOEBA14 water; Figure S10: Dipole histograms of AMOEBA14 water; Figure S11: Individual contributions to the THz spectrum of IPOl-0.13 water; Figure S12: Dipole histograms of IPOL-0.13 water; Figure S13: Individual contributions to the THz spectrum of OPC3-pol water; Figure S14: Dipole histograms of OPC3-pol water; Figure S15: The unconstrained model in a dash dotted blue line was tested against the constrained model in dark blue and the experiment. References [83,84,85,86,87] are cited in the Supplementary Materials.
