# Supplementary material for: Insights from the Absorption Coefficient for the Development of Polarizable (Multipole) Force Fields
Source: Molecules. 2025 Jul 11;30(14):2941. doi: 10.3390/molecules30142941 (PMC12300768; doi:10.3390/molecules30142941)
Supplement: Supplementary file 1 [file molecules-30-02941-s001.zip › molecules-3682843-supplementary.pdf]

Supplementary Materials: Insights from the Absorption Coefficient for the Development of Polarizable (Multipole) Force Fields

Marion Sappl,[1,2] András Szabadi,[1,2] Philipp Honegger,[3] Franziska König,[1] Othmar Steinhauser[1] and Christian Schröder[1*]

1. Methods

1.1. Computational Details

Initial configurations of 4000 molecules of either SPC/E [30], SWM4-NDP [36], IPOL-0.13 [40] or OPC3-pol [39] and 500 molecules of AMOEBA14 [42] water were generated by PACKMOL [83]. The SPC/E and SWM4 configurations were minimized 100 steps of steepest descent and 20 steps of adopted base Newton-Raphson minimization in CHARMM [84]. Additionally, 150 minimization steps running the L-BFGS [85] algorithm were carried out for all water models in OPENMM [57]. Subsequently, 10 ns (SPC/E and SWM4) or 5 ns (IPOL-0.13 and OPC3-pol) of equilibration were carried out in OPENMM in the npT ensemble using a Monte Carlo barostat at a pressure of 1 atm and a temperature of 300 K. The integration time step was set to 0.5 fs using the velocity-Verlet integrator for SWM4 and SPC/E water, and the Nose-Hoover integrator for IPOL-0.13 and OPC3-pol, with OPC3-pol using a time step of 0.25 fs due to unstable simulations. The geometry of water molecules was kept rigid for trajectories with SPC/E, SWM4, POL-0.13 and OPC3-pol water models. AMOEBA was equilibrated using the same parameters, except that the MTSLangevin integrator was used and the time step was set to 2 fs and total equilibration time was 2 ns and the water model flexible. Coulombic electrostatic interactions were handled using the periodic mesh Ewald method with an error tolerance of 0.0005, Lennard-Jones terms were smoothly switched off at a distance of 12 Å for SWM4, SPC/E, IPOL-0.13 and OPC3-pol systems, and 10 Å for AMOEBA due to size limitation of the system. In the polarizable SWM4 and IPOL-0.13 trajectories, small Drude oscillators with a mass of 0.4 u were attached to oxygen atoms. Their temperature was set to 1 K. For the OPC3-pol model, the Drude particle mass was set to 7.9995 u, and the Drude temperature was also maintained at 1 K. In all Drude simulations, a Drude hard wall constraint of 0.2 Å was applied to limit Drude displacements. The relationship between polarizability α, Drude charge q_D and force constant k_D is

$$q_D = \sqrt{4\pi\epsilon_0 \, k_D \, \alpha}. \tag{S1}$$

The polarizability of SWM4-NDP water is 0.978 25 Å^3, corresponding to a Drude charge of −1.716 36 e. Increasing the polarizability by 10 % results in a Drude charge of −1.800 16 e, while a 20 % increase yields −1.880 21 e. The Drude charge of IPOL-0.13 was set to −2.945 e, which is considerably lower than the suggested value of 1000 e [40] but higher than all other tested models. The polarizability of IPOL-0.13 was kept at 1.44 Å^3, consistent with the original model. The Drude charge of OPC3-pol is 1.32 e with a polarizability of 1.16 Å^3.

Finally, production runs of 20 ps (SPC/E), 40 ps (SWM4, IPOL-0.13, OPC3-pol), and 60 ps (AMOEBA) were carried out in the nVT ensemble with an integration time step of 0.2 fs (SWM4, SPC/E, IPOL-0.13 and OPC3-pol) and 0.1 fs (AMOEBA), respectively. While SWM4, IPOL-0.13, and OPC3-pol required about 5–6 hours for simulations and post-production analysis, AMOEBA required a similar amount of time but for a much smaller system size. The main limitation of AMOEBA systems, however, was the complexity of calculating dipole moments, as it required four times the data for each simulation time step, making RAM the primary limiting resource. The SPC/E simulation itself was the

shortest, but calculating the induced dipole moments during post-production took the longest, requiring nearly 35 hours in total, including analysis.

1.2. Post-production analysis

The coordinates, velocities, and atomic dipole moments were extracted from the OPENMM trajectories with programs written in Python3 using the MDANALYSIS module [86,87].

In the case of the non-polarizable SPC/E model, the induced dipole of each water molecule was computed in a self-consistent manner. Each molecular induced dipole moment $\vec{\mu}_i^{\text{ind}}(t)$ is inherently influenced by all other molecular induced dipole moments $\vec{\mu}_j^{\text{ind}}(t)$ and usually converged after four iterative steps. For reasons of computational performance, the corresponding algorithm was implemented in C++ functions, which were called by wrapper functions written in Cython. Nevertheless, the computational effort of this post-production run is already slightly higher than that of producing a polarizable trajectory. However, the so-obtained induced dipoles may be necessary to compute the peak at 200 cm^{-1}, although polarizable forces have never acted on the oxygen and hydrogen atoms. This way, we discriminate between the instantaneous response of the post-production induced dipole moments to the local electric field and the polarizable forces during the trajectory production altering Coulomb interactions.

In the SWM4 water, the polarizable forces already act on the atoms during the trajectory production. The computation of the induced dipole moments is straightforward for SWM4-NDP, as the coordinates of all atoms and Drude particles are already part of the trajectory.

The computational costs of the AMOEBA water simulations are the highest for the water models employed in this study. In addition to the more costly calculation of the multipole interactions, the AMOEBA water needed two additional logs of atomic dipole moments in OPENMM, one for the permanent $\vec{\mu}_{i\beta}^{\text{perm}}(t)$ and one for the induced dipole moments $\vec{\mu}_{i\beta}^{\text{ind}}(t)$. Printing the respective dipole moments at each time step decelerates the trajectory production even further. Furthermore, reading the necessary files for analysis requires significant memory, limiting the analysis to smaller systems or shorter time spans.

2. Decomposed spectra and dipole moment distributions

The following section shows the decomposed spectra. The top panel show the total spectra, the middle panel show the permanent dipole contributions, and the bottom panel show the induced dipole contributions. There is a cross-correlation of induced and permanent dipole moments, which is included once each in the permanent and the induced contribution due to the chosen decomposition.

Given the numerous possible contributing factors, we selected specific contributions that allow us to reproduce the spectra while still identifying which interactions are responsible for the observed signals. The spectra were decomposed in total, permanent and

induced contributions. Each of these were then decomposed in self, cross and total terms. The spectra were calculated from the following time correlation functions:

$$C^{tot}_{tot}(t) = \langle \vec{J}_D(0) \cdot \vec{J}_D(t) \rangle \tag{S2}$$

$$C^{tot}_{self}(t) = \langle \sum_i \frac{\vec{\mu}_i(0)}{dt} \cdot \frac{\vec{\mu}_i(t)}{dt} \rangle \tag{S3}$$

$$C^{tot}_{cross}(t) = \langle \sum_i \sum_{j \neq i} \frac{\vec{\mu}_i(0)}{dt} \cdot \frac{\vec{\mu}_j(t)}{dt} \rangle \tag{S4}$$

$$C^{perm}_{tot}(t) = \langle \vec{J}^{perm}_D(0) \cdot \vec{J}_D(t) \rangle \tag{S5}$$

$$C^{perm}_{self}(t) = \langle \sum_i \frac{\vec{\mu}^{perm}_i(0)}{dt} \cdot \frac{\vec{\mu}_i(t)}{dt} \rangle \tag{S6}$$

$$C^{perm}_{cross}(t) = \langle \sum_i \sum_{j \neq i} \frac{\vec{\mu}^{perm}_i(0)}{dt} \cdot \frac{\vec{\mu}_j(t)}{dt} \rangle \tag{S7}$$

$$C^{ind}_{tot}(t) = \langle \vec{J}^{ind}_D(0) \cdot \vec{J}_D(t) \rangle \tag{S8}$$

$$C^{ind}_{self}(t) = \langle \sum_i \frac{\vec{\mu}^{ind}_i(0)}{dt} \cdot \frac{\vec{\mu}_i(t)}{dt} \rangle \tag{S9}$$

$$C^{ind}_{cross}(t) = \langle \sum_i \sum_{j \neq i} \frac{\vec{\mu}^{ind}_i(0)}{dt} \cdot \frac{\vec{\mu}_j(t)}{dt} \rangle \tag{S10}$$

$$\tag{S11}$$

2.1. SPCE

2.1.1. Spectra

The decomposed spectra of SPC/E are depicted in Fig. S1. The intensity of the spectra of SPC/E water is too high compared to the experiment. However, this is expected as the intensity is dependent on the charges of the atoms. SPC/E is non-polarizable, therefore the permanent dipole moment is already quite high compared to the other models, as there is no induced contribution in the standard model. The intensity of the spectrum calculated from the permanent dipole moments is about 6000 cm^{-1} at the maximum of the libration peak, which is similar to the experimental spectrum. When the polarizability is added, the total dipole moments are higher than anticipated leading to higher intensities in the total spectrum. Furthermore, the peak at 200 cm^{-1} is virtually solely caused by induced self terms in this model. This might be due to the polarizability being added post-production.

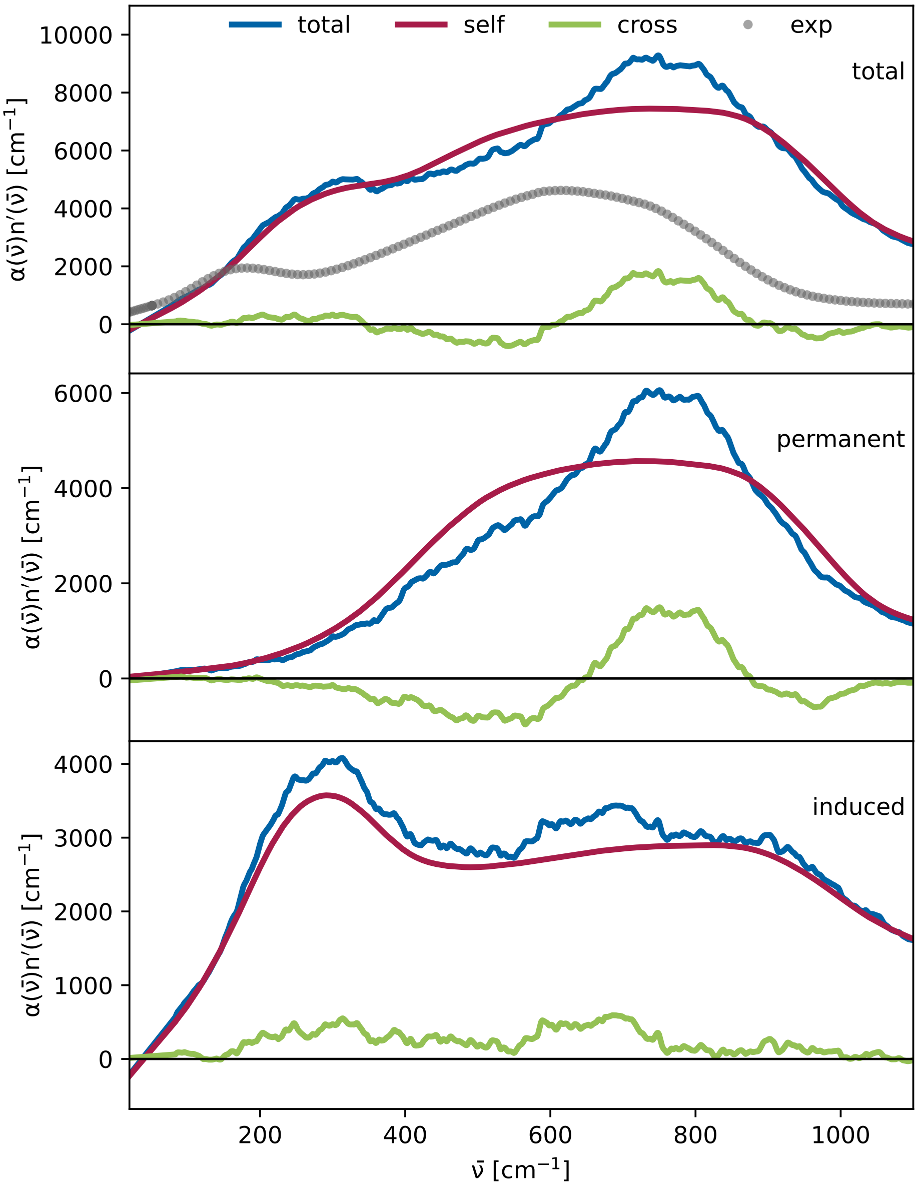

Figure S1. Individual contributions to the THz spectrum of SPC/E water.

2.1.2. Dipole histograms

The distributions of the dipole moments of SPC/E water are depicted in Fig. S2. SPC/E has a fixed geometry, therefore the absolute value of the permanent dipole moment is constant at 2.36 D. This leads to similar shaped dipole moment distributions of induced and total dipole moments. The shapes are similar, but are not necessarily the same, as the alignment of permanent and induced dipole moments can deviate from parallel alignment, leading to more complex relations between absolute values of permanent, induced and total dipole moments. Overall, the distributions of the induced and total dipole moments of SPC/E are right skewed, with a tendency to higher values compared to all other water models.

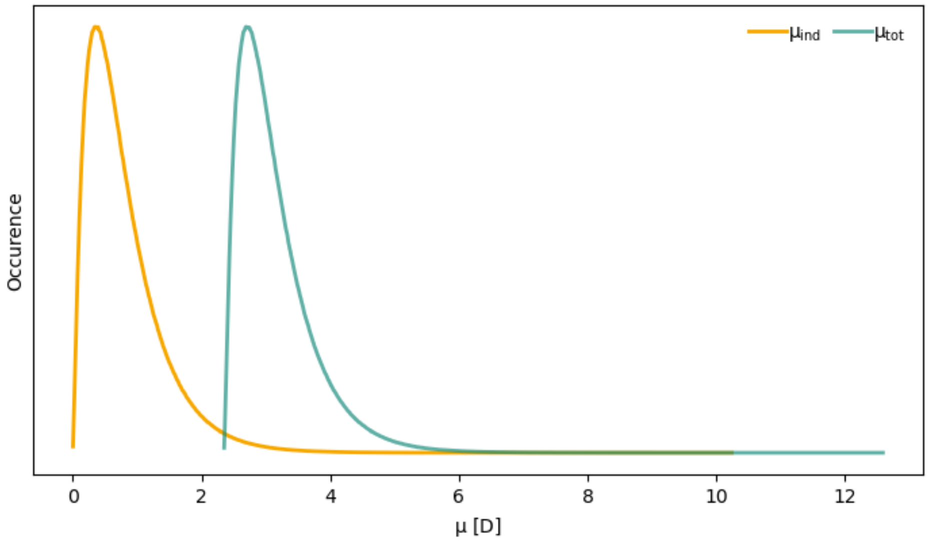

Figure S2. Dipole histograms of SPC/E water.

2.2. *SWM4-NDP*

2.2.1. Spectra

The decomposed spectra for SWM4-NDP are depicted in Fig. S3.The intensity of the libration peak in the total spectrum is close to the experiment, but the model underestimates the translation peak at 200 cm^{-1}, so that there is only a shoulder. The self terms of the permanent dipole moments are the main contributors to the libration peak, which is consistent with the other models. The main contribution to the translation peak is from induced dipole moments, with significant contributions from cross terms.

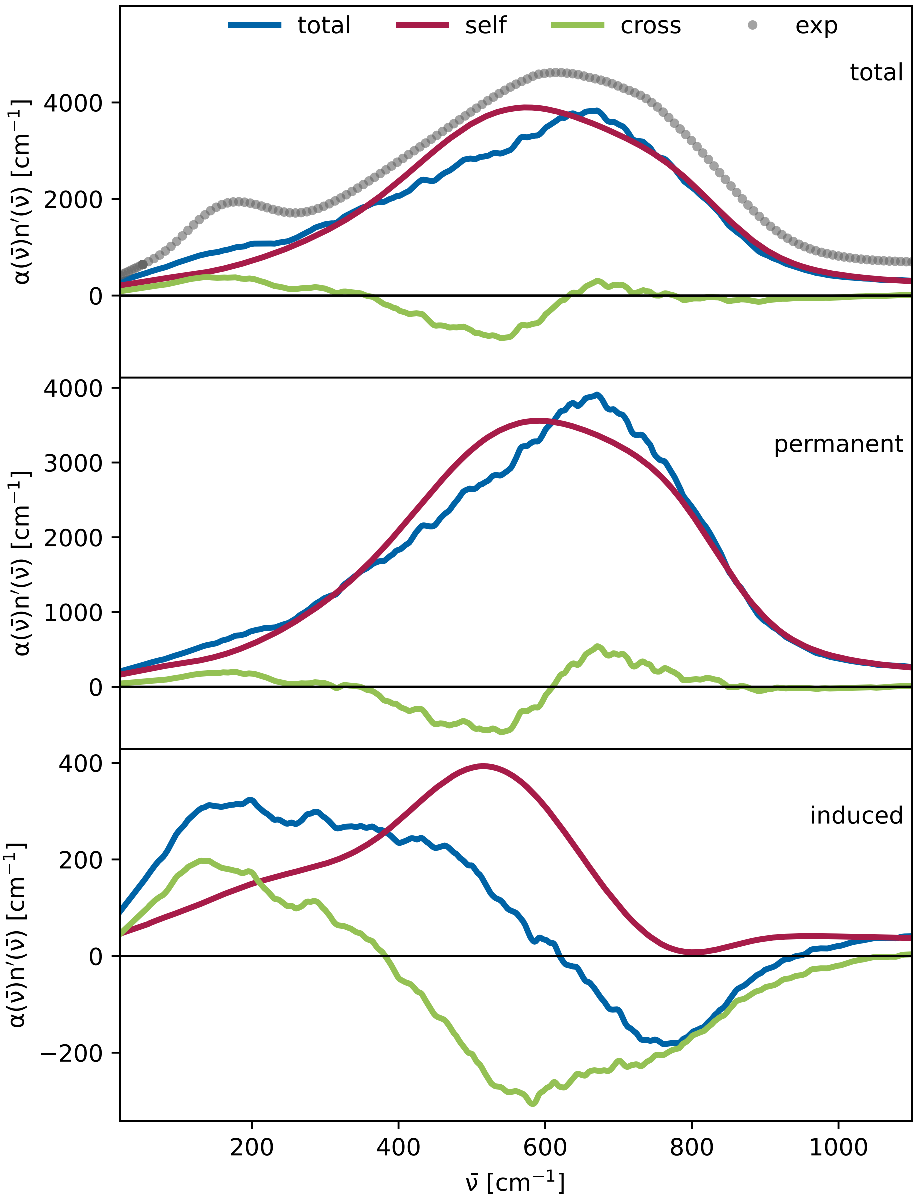

Figure S3. Individual contributions to the THz spectrum of SWM4-NDP water.

2.2.2. Dipole histograms

The dipole histograms for the SWM4-NDP water model are depicted in Fig. S4. The SWM4-NDP water model is a rigid water model with a permanent dipole moment of 1.85 D. This leads to similar shapes of induced and permanent dipole moment histograms. The dipole moment histograms are bell shaped.

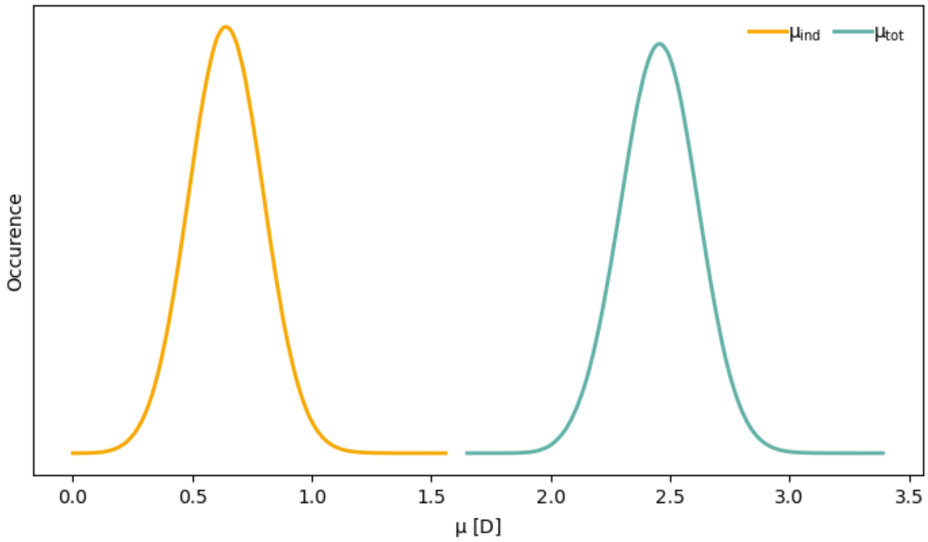

Figure S4. Dipole histograms of SWM4-NDP water.

2.3. *SWM4-NDP + 10 % polarization*

2.3.1. Spectra

The decomposed spectra for SWM4-NDP with 10 % polarizability are depicted in Fig. S5. Even though the polarizability is increased, the 200 cm^{-1} is not more pronounced than in the original model. The self terms of the permanent dipole moments are the main contributors to the libration peak, which is consistent with the other models. The main contribution to the translation peak is from induced dipole moments, with significant contributions from cross terms.

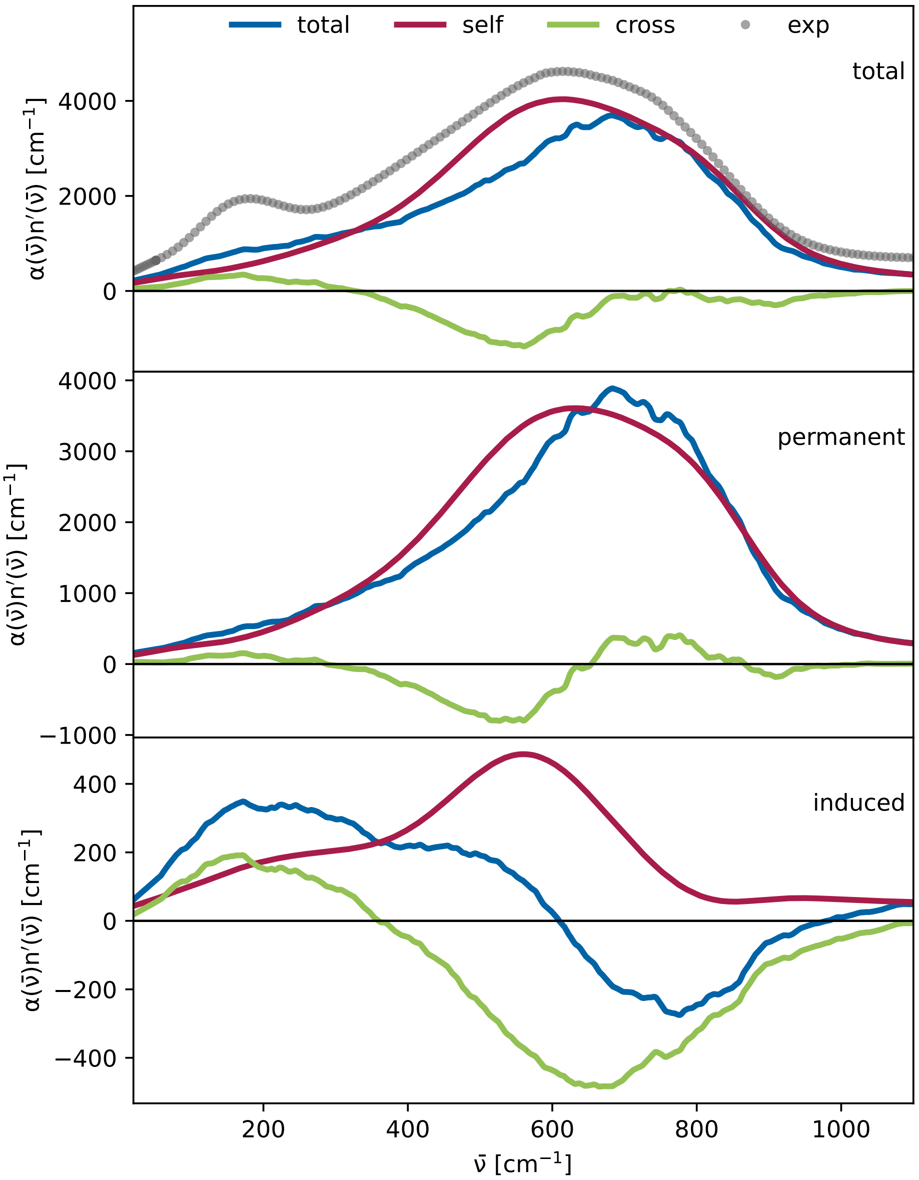

Figure S5. Individual contributions to the THz spectrum of SWM4-NDP water with 10 % increased polarizability.

2.3.2. Dipole histograms

The dipole histograms for the SWM4-NDP with 10 % polarizability water model are depicted in Fig. S4. This model has the same permanent dipole moment as the standard SWM4-NDP model. This leads to similar dipole moment histograms. Due to the increased polarizability, the values of the dipole moments are larger than the standard model's.

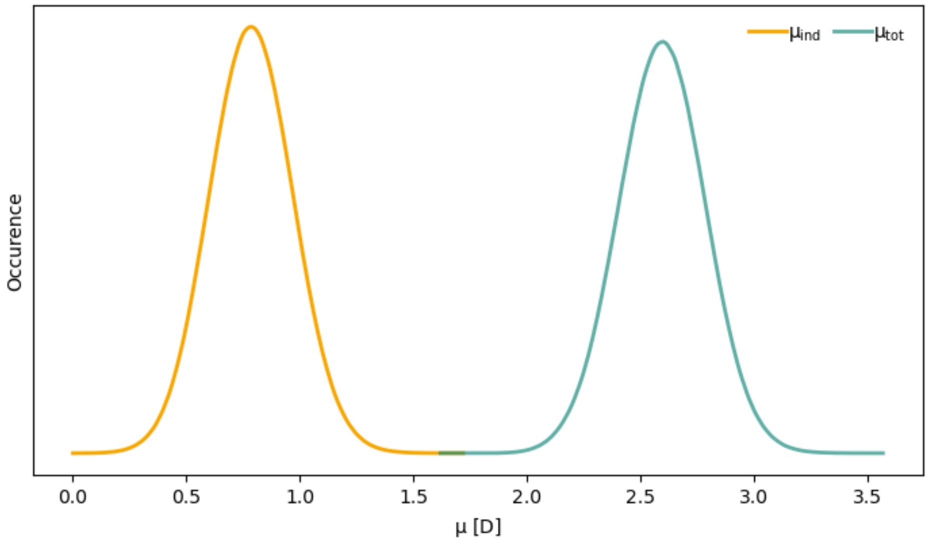

Figure S6. Dipole histograms of SWM4-NDP water with 10 % increased polarizability.

2.4. SWM4-NDP + 20 % polarization

2.4.1. Spectra

The decomposed spectra for SWM4-NDP with 20 % polarizability are depicted in Fig. S7. The intensity of the libration peak in the total spectrum is close to the experiment, but the model underestimates the translation peak at 200 cm^{-1}, so that there is only a shoulder. The self terms of the permanent dipole moments are the main contributors to the libration peak, which is consistent with the other models. The main contribution to the translation peak is from induced dipole moments, with significant contributions from cross terms.

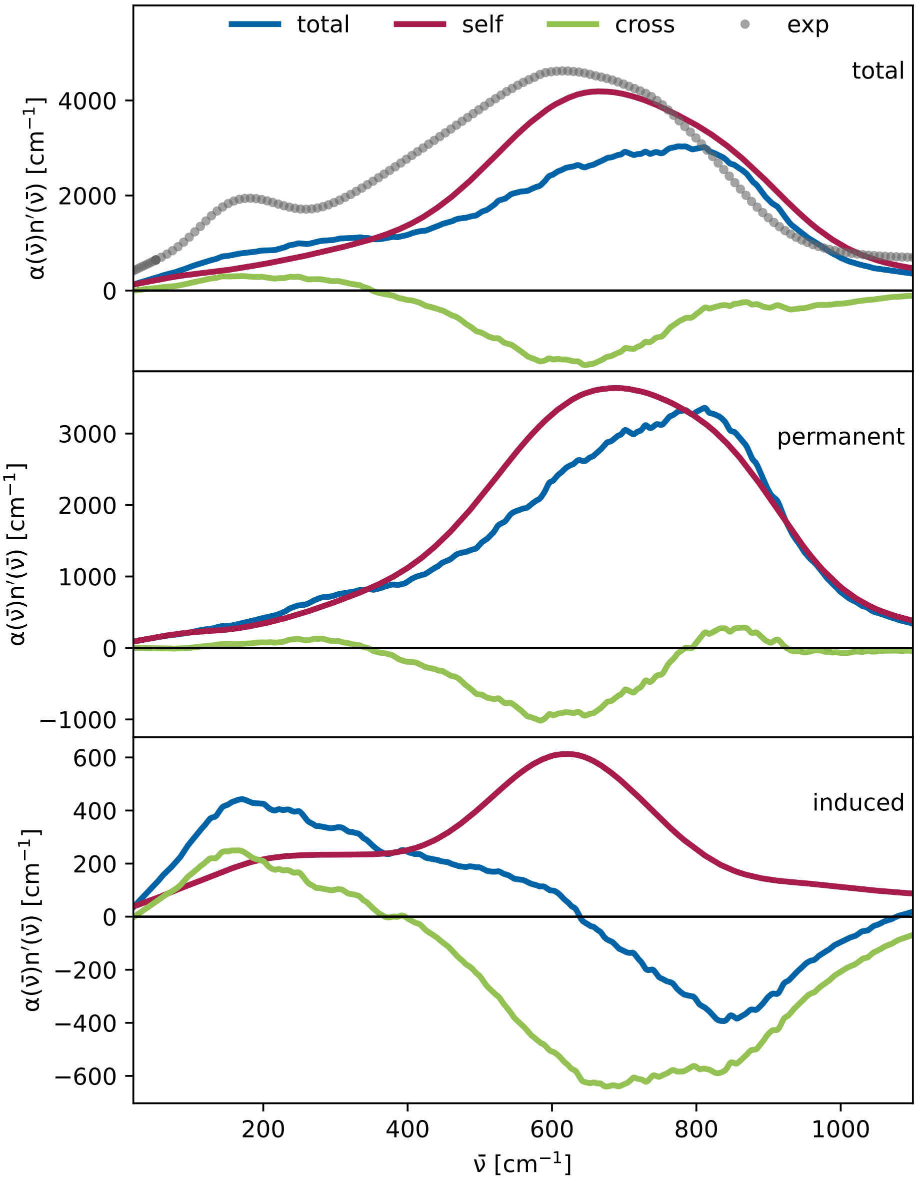

Figure S7. Individual contributions to the THz spectrum of SWM4-NDP water with 20 % increased polarizability.

2.4.2. Dipole histograms

The SWM4-NDP with 20 % polarizability has the same permanent dipole moment as the standard SWM4-NDP model. Due to the increased polarizability, the values of the dipole moments are shifted to even higher values than the standard model and the one with 10 % increased polarizability.

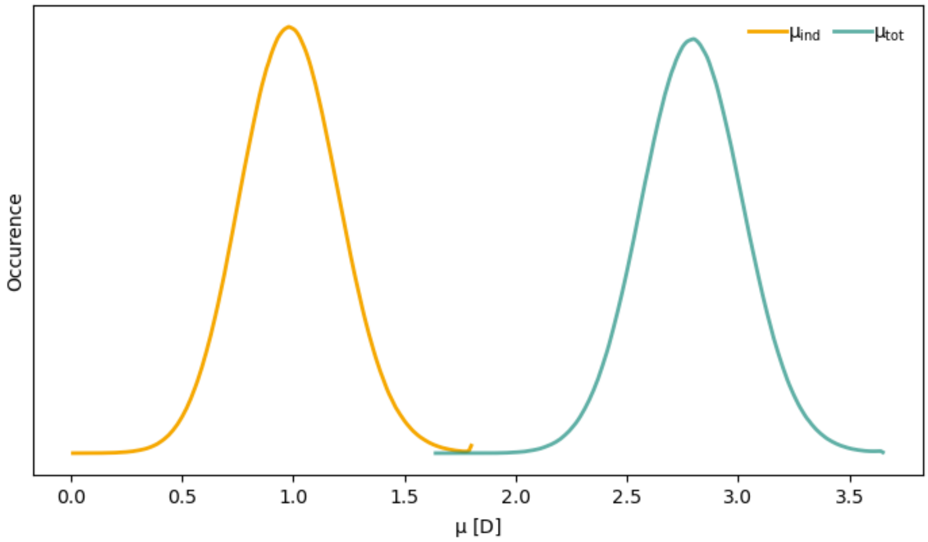

Figure S8. Dipole histograms of SWM4-NDP water with 20 % increased polarizability.

2.5. AMOEBA14

2.5.1. Spectra

The decomposed spectra of the AMOEBA14 water model are depicted in Fig. S9. Similar to the other water models, the libration peak at 600 cm^{-1} is mainly due to permanent self terms. The induced contributions to the vibration peak at 200 cm^{-1} is more pronounced than in all other water models making it the most suitable model that we investigated. It is also the only model that has mathematical atomic dipole moments on all atoms, while all other water models were made polarizable by making the oxygen atom polarizable. Furthermore, the AMOEBA14 water model was the only flexible water model in this study.

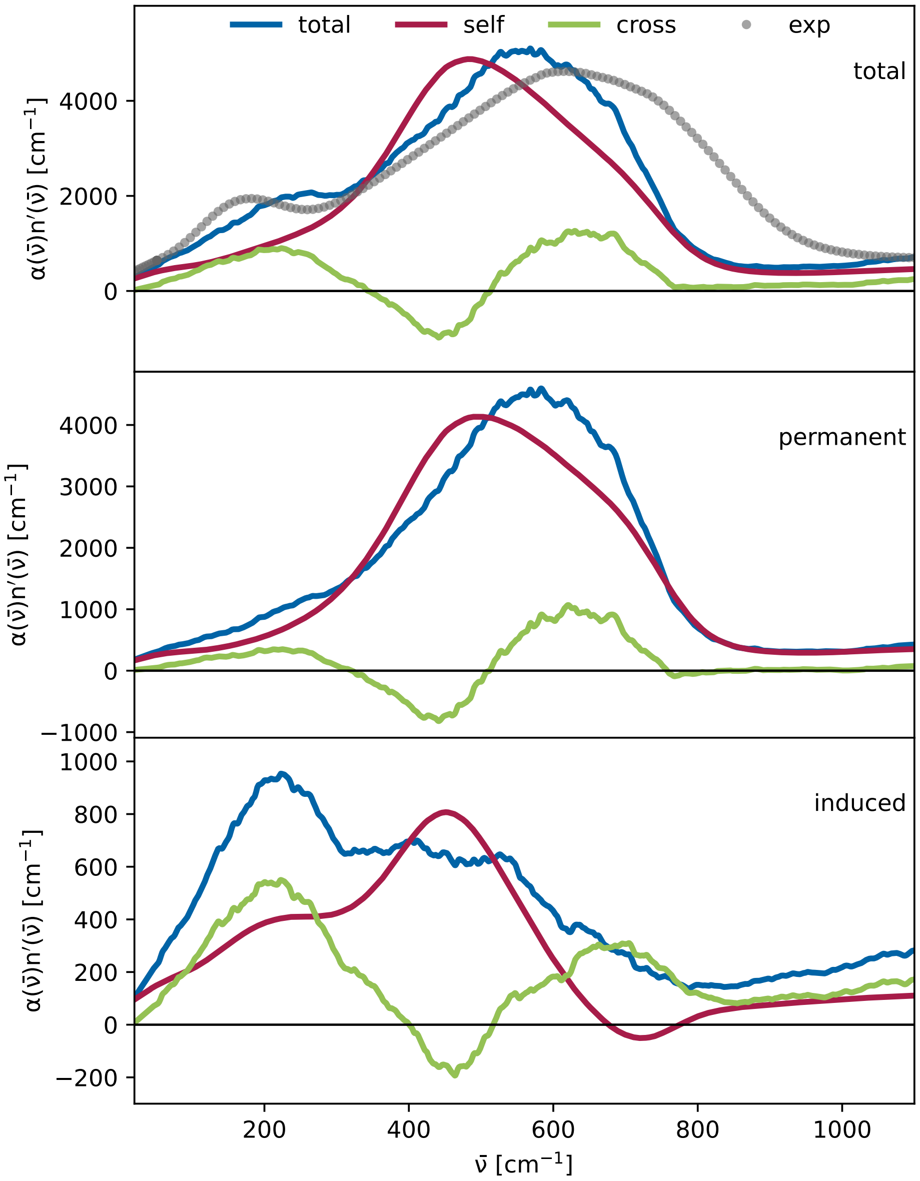

Figure S9. Individual contributions to the THz spectrum of AMOEBA14 water.

2.5.2. Dipole histograms

The dipole histograms of the AMOEBA14 water model are depicted in Fig. S10. Due to the flexible geometry, the total dipole moment exhibits a broadened bell curve compared to the induced dipole moment histogram. The shapes are bell curves.

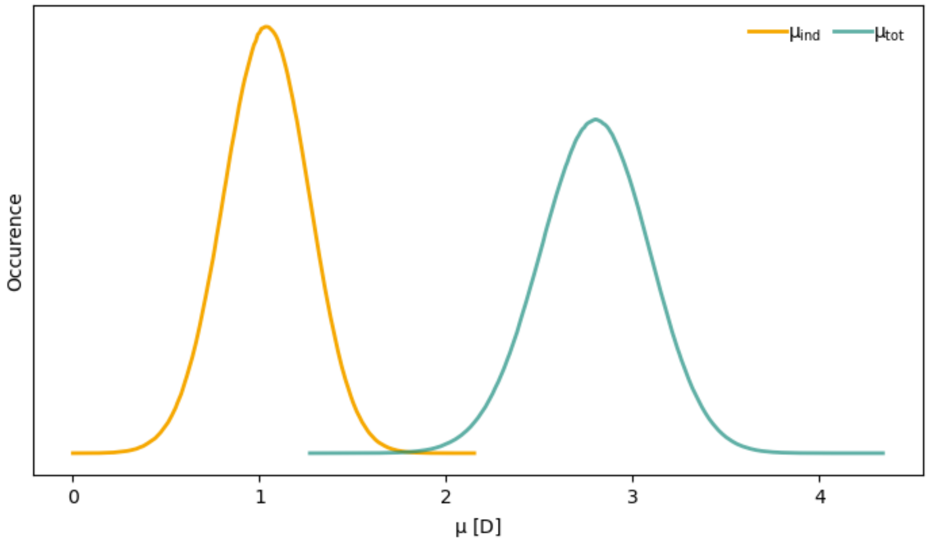

Figure S10. Dipole histograms of AMOEBA14 water.

2.6. IPOL-0.13

2.6.1. Spectra

The decomposed spectra of the IPOL-0.13 water model are depicted in Fig. S11. In general, the compositions are very similar to those of the SWM4-NDP water model.

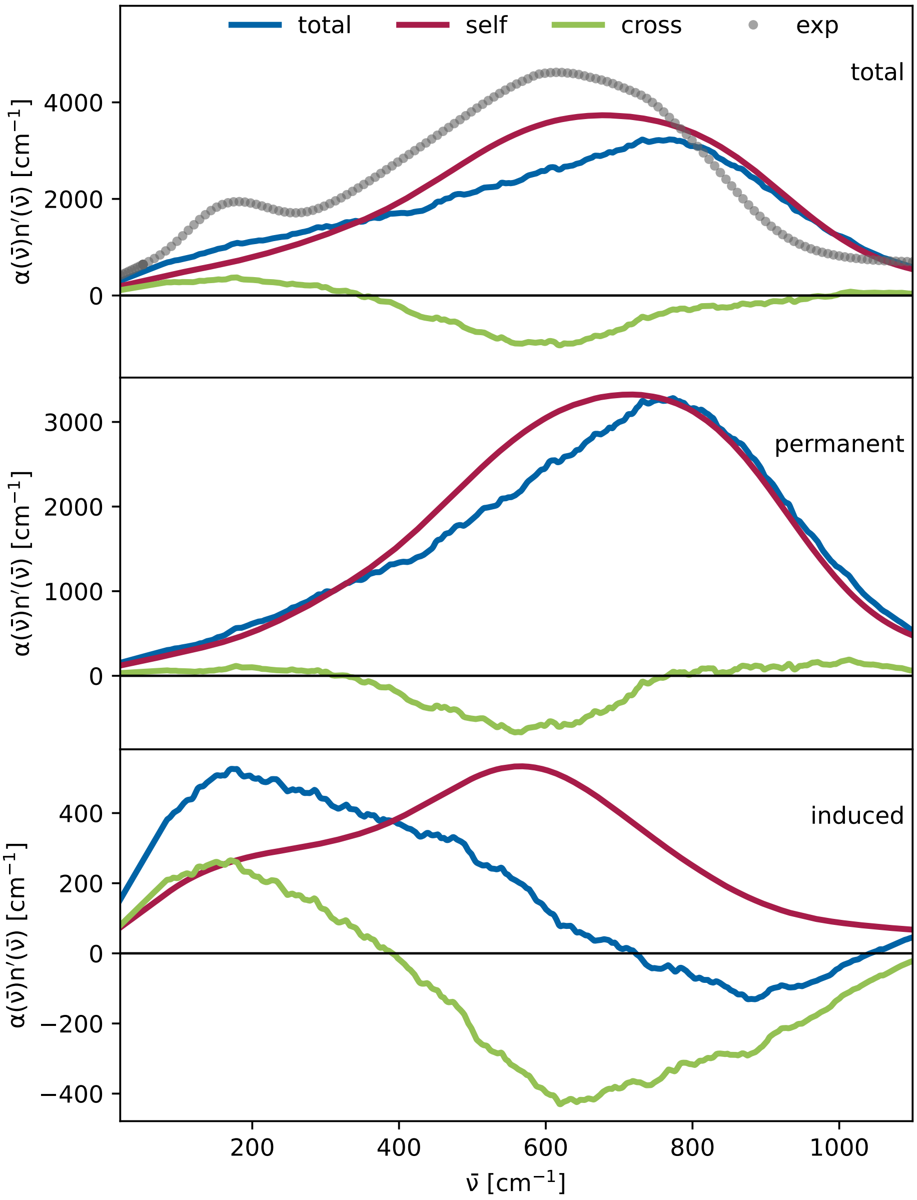

Figure S11. Individual contributions to the THz spectrum of IPOl-0.13 water.

2.6.2. Dipole histograms

The dipole histograms of the IPOL-0.13 water model are depicted in Fig. S12. The water model is rigid, leading to similar shapes for the induced and total dipole moment histograms. The shapes of the dipole moment histograms are bell curves.

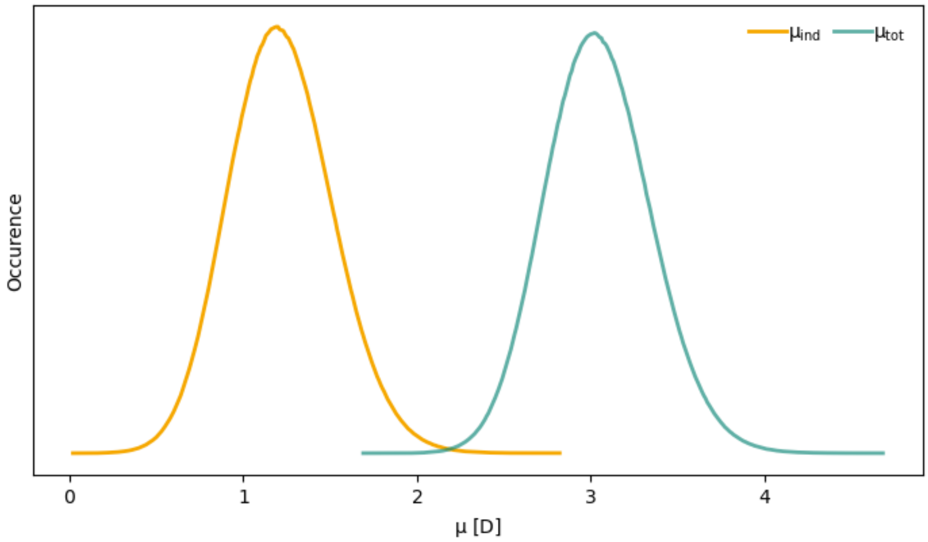

Figure S12. Dipole histograms of IPOL-0.13 water.

2.7. *OPC3-pol*

2.7.1. Spectra

The decomposed spectra of the OPC3-pol water model are depicted in Fig. S13. The peak maxima are shifted to higher frequencies than in the experimental spectra. Furthermore, the libration peak is narrower than in the other water models. Other than that, the general contributions are similar to those of the other Drude-polarizable water models.

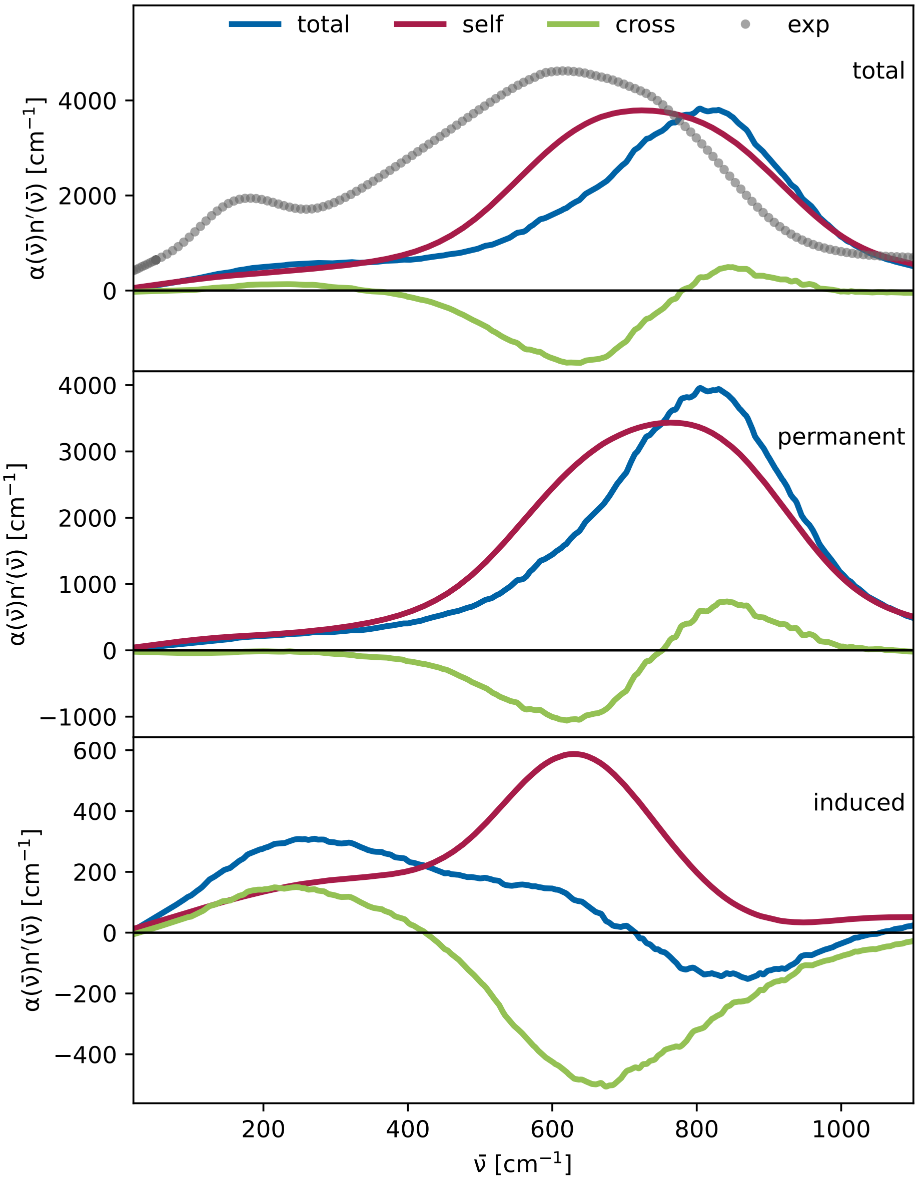

Figure S13. Individual contributions to the THz spectrum of OPC3-pol water.

2.7.2. Dipole histograms

The dipole histograms of the OPC3-pol water model are depicted in Fig. S14. The dipole histograms are left skewed, which is likely due to the Drude hard wall constraint.

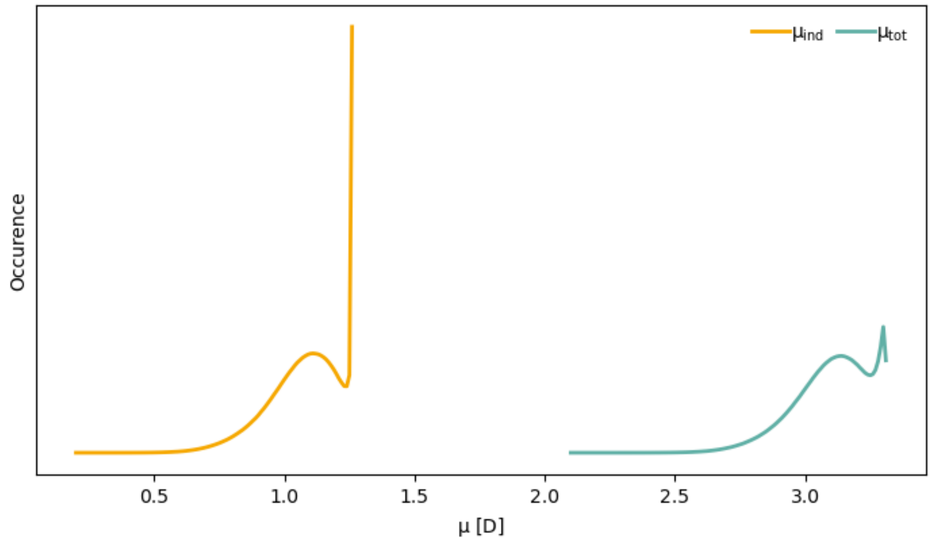

Figure S14. Dipole histograms of OPC3-pol water.

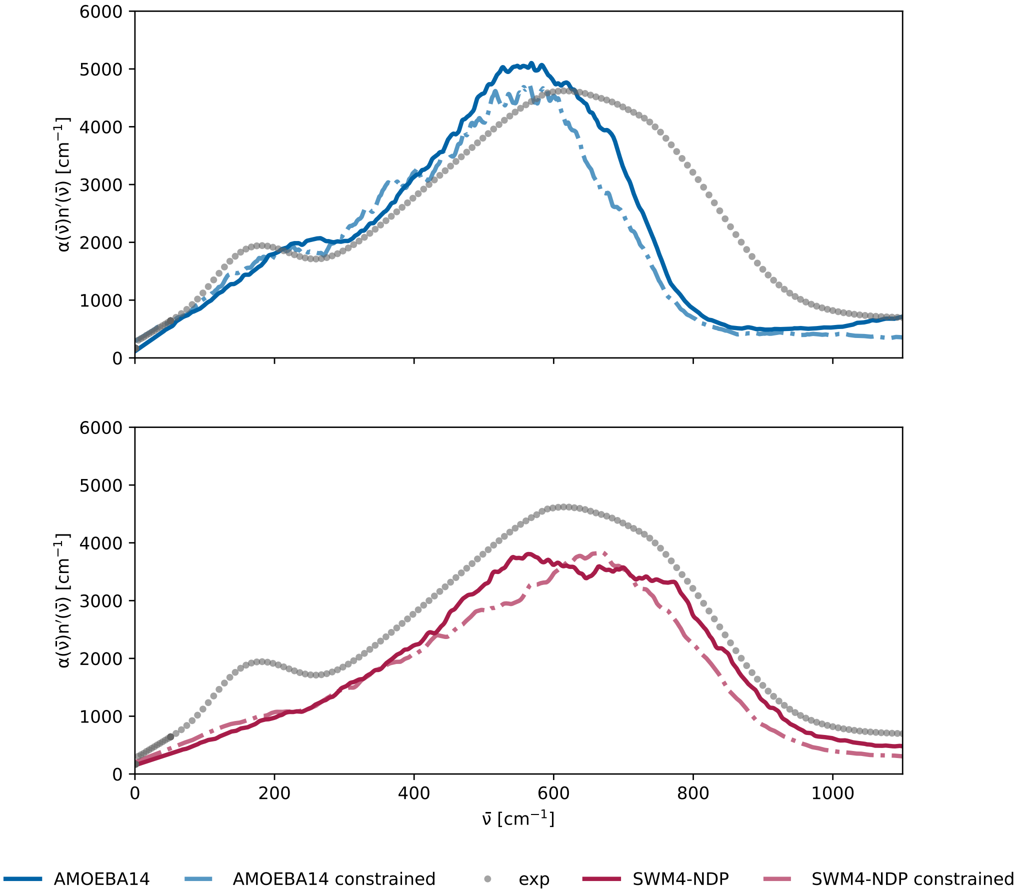

Figure S15. The unconstrained model in a dash dotted blue line was tested against the constrained model in dark blue and the experiment.

3. Influence of angle- and hydrogen bond constraints

We tested two water models, SWM4-NDP and AMOEBA14, in constrained and unconstrained simulations. The spectra are depicted in Fig. S15. The AMOEBA14 water model is a flexible model, therefore the unconstrained version is the original model. We chose to use intramolecular hydrogen bond length constraints and the SHAKE algorithm to keep hydrogen bond lengths and HOH-angles constant. Making the AMOEBA14 model rigid had little influence on the spectrum. Next, we tested the SWM4-NDP water model, comparing an unconstrained version to the constrained version, where bond lengths and angles involving hydrogen atoms were fixed using SHAKE. Since the SWM4-NDP water model is designed to be rigid, the constrained version represents the standard implementation. In this case, while the libration peak broadened slightly, the peak at 200 cm^{-1} was even smaller than in the unconstrained model. Overall, the differences between constrained and unconstrained simulations for both models were minimal. Based on these results, we conclude that allowing molecular bond and angle vibrations by removing constraints does not significantly influence the terahertz spectrum.

1. Wulf, A.; Fumino, K.; Ludwig, R.; Taday, P.F. Combined THz, FIR and Raman Spectroscopy Studies of Imidazolium-Based Ionic Liquids Covering the Frequency Range 2–300 cm^{-1}. *Chem. Phys. Chem.* **2010**, *11*, 349–353.
2. Zasetsky, A.Y.; Lileev, A.S.; Lyashchenko, A.K. Molecular dynamic simulations of terahertz spectra for water-methanol mixtures. *Mol. Phys.* **2010**, *108*, 649–656.

3. Cherkasova, O.; Nazarov, M.; Konnikova, M.; Shkurinov, A. THz spectroscopy of bound water in glucose: Direct measurements from crystalline to dissolved state. *J. Infrared Millim.* **2020**, *41*, 1057–1068.
4. Ruiz-Barragan, S.; Sebastiani, F.; Schienbein, P.; Abraham, J.; Schwaab, G.; Nair, R.R.; Havenith, M.; Marx, D. Nanoconfinement effects on water in narrow graphene-based slit pores as revealed by THz spectroscopy. *Phys. Chem. Chem. Phys.* **2022**, *24*, 24734–24747.
5. Pyne, P.; Mahanta, D.D.; Gohil, H.; Prabhu, S.; Mitra, R.K. Correlating solvation with conformational pathways of proteins in alcohol–water mixtures: A THz spectroscopic insight. *Phys. Chem. Chem. Phys.* **2021**, *23*, 17536–17544.
6. Fei, S.; Hsu, W.L.; Delaunay, J.J.; Daiguji, H. Molecular dynamics study of water confined in MIL-101 metal–organic frameworks. *J. Chem. Phys.* **2021**, *154*, 144503.
7. Sega, M.; Schröder, C. Dielectric and terahertz spectroscopy of polarizable and nonpolarizable water models: A comparative study. *J. Phys. Chem. A* **2015**, *119*, 1539–1547.
8. Grechko, M.; Hasegawa, T.; D'Angelo, F.; Ito, H.; Turchinovich, D.; Nagata, Y.; Bonn, M. Coupling between intra- and intermolecular motions in liquid water revealed by twodimensional terahertz-infrared-visible spectroscopy. *Nat. Commun.* **2018**, *9*, 885.
9. Novelli, F.; Pestana, L.R.; Bennett, K.C.; Sebastiani, F.; Adams, E.M.; Stavrias, N.; Ockelmann, T.; Colchero, A.; Hoberg, C.; Schwaab, G.; et al. Strong Anisotropy in Liquid Water upon Librational Excitation Using Terahertz Laser Fields. *J. Phys. Chem. B* **2020**, *124*, 4989–5001.
10. Novelli, F.; Hoberg, C.; Adams, E.M.; Klopf, J.M.; Havenith, M. Terahertz pump–probe of liquid water at 12.3 THz. *Phys. Chem. Chem. Phys.* **2022**, *24*, 653–665.
11. Laage, D.; Elsaesser, T.; Hynes, J.T. Water Dynamics in the Hydration Shells of Biomolecules. *Chem. Rev.* **2017**, *117*, 10694–10725.
12. Williams, G.P. Filling the THz gap—high power sources and applications. *Rep. Prog. Phys.* **2005**, *69*, 301.
13. Perakis, F.; De Marco, L.; Shalit, A.; Tang, F.; Kann, Z.R.; Kühne, T.D.; Torre, R.; Bonn, M.; Nagata, Y. Vibrational Spectroscopy and Dynamics of Water. *Chem. Rev.* **2016**, *116*, 7590–7607.
14. Hasted, J.B.; Husain, S.K.; Frescura, F.A.M.; Birch, J.R. Far-infrared absorption in liquid water. *Chem. Phys. Lett.* **1985**, *118*, 622–625.
15. Chakraborty, S.; Sinha, S.K.; Bandyopadhyay, S. Low-Frequency Vibrational Spectrum of Water in the Hydration Layer of a Protein: A Molecular Dynamics Simulation Study. *J. Phys. Chem. B* **2007**, *111*, 13626–13631.
16. Heyden, M.; Havenith, M. Combining THz spectroscopy and MD simulations to study protein-hydration coupling. *Methods* **2010**, *52*, 74–83.
17. Inagaki, T.; Hatanaka, M.; Saito, S. Anisotropic and Finite Effects on Intermolecular Vibration and Relaxation Dynamics: Low-Frequency Raman Spectroscopy of Water Film and Droplet on Graphene by Molecular Dynamics Simulations. *J. Phys. Chem. B* **2023**, *127*, 5869–5880.
18. Torii, H. Simulations of the THz spectrum of liquid water incorporating the effects of the intermolecular charge fluxes through hydrogen bonds. *AIP Conf. Proc.* **2015**, *1702*, 090043.
19. Chen, W.; Sharma, M.; Resta, R.; Galli, G.; Car, R. Role of dipolar correlations in the infrared specra of water and ice. *Phys. Rev. B* **2008**, *77*, 245114.
20. Heyden, M.; Sun, J.; Funkner, S.; Mathias, G.; Forbert, H.; Havenith, M.; Marx, D. Dissecting the THz spectrum of liquid water from first principles via correlations in time and space. *Proc. Natl. Acad. Sci. USA* **2010**, *107*, 12068–12073.
21. Carlson, S.; Brünig, F.N.; Loche, P.; Bonthuis, D.J.; Netz, R.R. Exploring the absorption spectrum of simulated water from MHz to Infrared. *J. Phys. Chem. A* **2020**, *124*, 5599.
22. Madden, P.A.; Impey, R.W. On the infrared and Raman spectra of water in the region 5-250 cm^{-1}. *Chem. Phys. Lett.* **1986**, *123*, 502–506.
23. Demerdash, O.; Wang, L.P.; Head-Gordon, T. Advanced models for water simulations. *WIREs Comput Mol Sci* **2018**, *8*, e1355.
24. Babin, V.; Medders, G.R.; Paesani, F. Development of a "First Principles" Water Potential with Flexible Monomers. II: Trimer Potential Energy Surface, Third Virial Coefficient, and Small Clusters. *J. Chem. Theory Comput.* **2014**, *10*, 1599–1607.
25. Joerg, F.; Wieder, M.; Schröder, C. Protex—A Python utility for proton exchange in molecular dynamics simulations. *Front. Chem. Sec. Mol. Liq.* **2023**, *11*, 1140896.
26. Gődény, M.; Joerg, F.; Kovar, M.P.P.; Schröder, C. Updates to Protex for Simulating Proton Transfers in an Ionic Liquid. *J. Phys. Chem. B* **2024**, *128*, 3416–3426.
27. Sharma, M.; Resta, R.; Car, R. Intermolecular Dynamical Charge Fluctuations in Water: A Signature of the H-Bond Network. *Phys. Rev. Lett.* **2005**, *95*, 187401.

28. Sharma, D.; Das, B.; Chandra, A. Terahertz Spectrum of Water at Varying Temperatures from 260 to 340 K: Contributions from Permanent and Induced Dipole Correlations at Different Length Scales. *J. Phys. Chem. B* **2023**, *127*, 6714–6725.
29. Han, B.; Isborn, C.M.; Shi, L. Incorporating Polarization and Charge Transfer into a Point-Charge Model for Water Using Machine Learning. *J. Phys. Chem. Lett.* **2023**, *14*, 3869–3877.
30. Berendsen, H.J.C.; Grigera, J.R.; Straatsma, T.P. The Missing Term in Effective Pair Potentials. *J. Phys. Chem.* **1987**, *91*, 6269.
31. Abascal, J.L.F.; Vega, C. A general purpose model for the condensed phases of water: TIP4P/2005. *J. Chem. Phys.* **2005**, *123*, 234505.
32. Wu, Y.; Tepper, H.L.; Voth, G.A. Flexible simple point-charge water model with improved liquid-state properties. *J. Chem. Phys.* **2006**, *124*, 024503.
33. González, M.A.; Abascal, J.L.F. A flexible model for water based on TIP4P/2005. *J. Chem. Phys.* **2004**, *135*, 224516.
34. Hamm, P. 2D-Raman-THz spectroscopy: A sensitive test of polarizable water models. *J. Chem. Phys.* **2014**, *141*, 184201.
35. Shi, L.; Ni, Y.; Drews, S.E.P.; Skinner, J.L. Dielectric constant and low-frequency infrared spectra for liquid water and ice Ih within the E3B model. *J. Chem. Phys.* **2014**, *141*, 084508.
36. Lamoureux, G.; Harder, E.; Vorobyov, I.V.; Roux, B.; MacKerell, A.D. A polarizable model of water for molecular dynamics simulations of biomolecules. *Chem. Phys. Lett.* **2006**, *418*, 245–249.
37. Sidler, D.; Meuwly, M.; Hamm, P. An efficient water force field calibrated against intermolecular THz and Raman spectra. *J. Chem. Phys.* **2018**, *148*, 244504.
38. Tröster, P.; Lorenzen, K.; Schwörer, M.; Tavan, P. Polarizable Water Models from Mixed Computational and Empirical Optimization. *J. Phys. Chem. B* **2013**, *117*, 9486–9500.
39. Xiong, Y.; Izadi, S.; Onufriev, A.V. Fast Polarizable Water Model for Atomistic Simulations. *J. Chem. Theory Comput.* **2022**, *18*, 6324–6333.
40. Kolafa, J. A Polarizable Three-Site Water Model with Intramolecular Polarizability. *Collect. Czech. Chem. Commun.* **2008**, *73*, 507–517.
41. Liu, C.; Piquemal, J.P.; Ren, P. Implementation of Geometry-Dependent Charge Flux into the Polarizable AMOEBA+ Potential. *J. Phys. Chem. Lett.* **1957**, *11*, 419–426.
42. Laury, M.L.; Wang, L.P.; Pande, V.S.; Head-Gordon, T.; Ponder, J.W. Revised Parameters for the AMOEBA Polarizable Atomic Multipole Water Model. *J. Phys. Chem. B* **2015**, *119*, 9423–9437.
43. Wang, L.P.; Head-Gordon, T.; Ponder, J.W.; Ren, P.; Chodera, J.D.; Eastman, P.K.; Martinez, T.J.; Pande, V.S. Systematic Improvement of a Classical Molecular Model of Water. *J. Phys. Chem. B* **2013**, *117*, 9956–9972.
44. Liu, C.; Piquemal, J.P.; Ren, P. AMOEBA+ Classical Potential for Modeling Molecular Interactions. *J. Chem. Theory Comput.* **2019**, *15*, 4122–4139.
45. Hasegawa, T.; Tanimura, Y. A Polarizable Water Model for Intramolecular and Intermolecular Vibrational Spectroscopies. *J. Phys. Chem. B* **2011**, *115*, 5545–5553.
46. Liu, J.; Miller, W.H.; Fanourgakis, G.S.; Xantheas, S.S.; Imoto, S.; Saito, S. Insights in quantum dynamical effects in the infrared spectroscopy of liquid water from a semiclassical study with an ab initio-based flexible and polarizable force field. *J. Chem. Phys.* **2011**, *135*, 244503.
47. Fanourgakis, G.S.; Xantheas, S.S. Development of transferable interaction potentials for water. V. Extension of the flexible, polarizable, Thole-type model potential TTM3-F, v. 3.0 to describe the vibrational spectra of water clusters and liquid water. *J. Chem. Phys.* **2008**, *128*, 074506.
48. Torii, H. Cooperative Contributions of the Intermolecular Charge Fluxes and Intramolecular Polarizations in the Far-Infrared Spectral Intensities of Liquid Water. *J. Chem. Theory Comput.* **2014**, *10*, 1219–1227.
49. Medders, G.R.; Paesani, F. Infrared and Raman Spectroscopy of Liquid Water through "First-Principles" Many-Body Molecular Dynamics. *J. Chem. Theory Comput.* **2015**, *11*, 1145–1154.
50. Liu, H.; Wang, Y.; Bowman, J.M. Quantum calculations of the IR spectrum of liquid water using ab initio and model potential and dipole moment surfaces and comparison with experiment. *J. Chem. Phys.* **2015**, *142*, 194502.
51. Elton, D.C.; Fernández-Serra, M.V. Polar nanoregions in water: A study of the dielectric properties of TIP4P/2005, TIP4P/2005f and TTM3F. *J. Chem. Phys.* **2014**, *140*, 124504.
52. Stukan, M.R.; Asmadi, A.; Abdallah, W. Bulk properties of SWM4-NDP water model at elevated temperature and pressure. *J. Mol. Liq.* **2013**, *180*, 65–69.
53. Malmberg, C.; Maryott, A. Dielectric constant of water from 0 to 100 C. *J. Res. Natl. Inst. Stand. Technol.* **1956**, *56*, 1–8.

54. Esser, A.; Belsare, S.; Marx, D.; Head-Gordon, T. Mode specific THz spectra of solvated amino acids using the AMOEBA polarizable force field. *Phys. Chem. Chem. Phys.* **2017**, *19*, 5579–5590.
55. Case, D.A.; Aktulga, H.M.; Belfon, K.; Cerutti, D.S.; Cisneros, G.A.; Cruzeiro, V.W.D.; Forouzesh, N.; Giese, T.J.; Götz, A.W.; Gohlke, H.; et al. AmberTools. *J. Chem. Inf. Model* **2023**, *63*, 6183–6191.
56. Rackers, J.A.; Wang, Z.; Lu, C.; Laury, M.L.; Lagardere, L.; Schnieders, M.J.; Piquemal, J.P.; Ren, P.; Ponder, J. Tinker 8: Software Tools for Molecular Desig. *J. Chem. Theory Comput.* **2018**, *14*, 5273–5289.
57. Eastman, P.; Swails, J.; Chodera, J.D.; McGibbon, R.T.; Zhao, Y.; Beauchamp, K.A.; Wang, L.P.; Simmonett, A.C.; Harrigan, M.P.; Stern, C.D.; et al. OpenMM 7: Rapid development of high performance algorithms for molecular dynamics. *PLoS Comput. Biol.* **2017**, *13*, e1005659 .
58. Wooten, F. *Optical Properties of Solids*; Academic Press: New York, NY, USA; London, UK, 1972.
59. Schröder, C.; Steinhauser, O. Using fit functions in computational dielectric spectroscopy. *J. Chem. Phys.* **2010**, *132*, 244109.
60. Thomas, M.; Brehm, M.; Fligg, R.; Vöhringer, P.; Kirchner, B. Computing vibrational spectra from ab initio molecular dynamics. *Phys. Chem. Chem. Phys.* **2013**, *15*, 6608–6622.
61. Szabadi, A.; Doknic, A.; Netsch, J.; Palvögyi, A.M.; Steinhauser, O.; Schröder, C. Force field refinement for reproducing experimental infrared spectra of ionic liquids. *Phys. Chem. Chem. Phys.* **2023**, *25*, 19882–19890.
62. Mauger, N.; Plé, T.; Lagardère, L.; Huppert, S.; Piquemal, J.P. Improving Condensed-Phase Water Dynamics with Explicit Nuclear Quantum Effects: The Polarizable Q-AMOEBA Force Field. *J. Phys. Chem. B* **2022**, *126*, 8813–8826.
63. Flór, M.; Wilkins, D.M.; de la Puente, M.; Laage, D.; Cassone, G.; Hassanali, A.; Roke, S. Dissecting the hydrogen bond network of water:Charge transfer and nuclear quantum effects. *Science* **2024**, *386*, 1110.
64. Machida, M.; Kato, K.; Shiga, M. Nuclear quantum effects of light and heavy water studied by all-electron first principles path integral simulations. *J. Chem. Phys.* **2017**, *148*, 102324.
65. Stone, A.J. *The Theory of Intermolecular Forces*; Clarendon Press: Oxford, UK, 1996.
66. Devereux, M.; Pezzella, M.; Raghunathan, S.; Meuwly, M. Polarizable Multipolar Molecular Dynamics Using Distributed Point Charges. *J. Chem. Theory Comput.* **2020**, *16*, 7267–7280.
67. Gray, C.G. Spherical tensor approach to multipole expansions. I. Electrostatic interaction. *Can. J. Phys.* **1975**, *54*, 505–512.
68. Pan, C.; Liu, C.; Peng, J.; Ren, P.; Huang, X. Three-site and five-site fixed-charge water models compatiblewith AMOEBA force field. *J. Comput. Chem.* **2020**, *41*, 1034–1044.
69. Ren, P.Y.; Ponder, J.W. Polarizable Atomic Multipole Water Model for Molecular Mechanics Simulation. *J. Phys. Chem. B* **2003**, *107*, 5933–5947.
70. Torabifard, H.; Starovoytov, O.N.; Ren, P.; Cisneros, G.A. Development of an AMOEBA water model using GEM distributed multipoles. *Theor. Chem. Acc.* **2015**, *134*, 101.
71. Das, A.K.; Demerdash, O.N.; Head-Gordon, T. Improvemnets to the AMOEBA Force Field by Introducing Anisotropic Atomic Polarizability of the Water Molecule. *J. Chem. Theory Comput.* **2018**, *14*, 6722–6733.
72. Rick, S.W.; Stuart, S.J.; Berne, B.J. Dynamical fluctuating charge force fields: Application to liquid water. *J. Chem. Phys.* **1994**, *101*, 6141–6156.
73. Rick, S.W.; Berne, B.J. Free Energy of the Hydrophobic Interaction from Molecular Dynamics Simulations: The Effects of Solute and Solvent Polarizability. *J. Phys. Chem. B* **1997**, *101*, 10488–10493.
74. Rick, S.W. Simulations of ice and liquid water over a range of temperatures using the fluctuating charge model. *J. Chem. Phys.* **2001**, *114*, 2276–2283.
75. Vácha, R.; Marsalek, O.; Willard, A.P.; Bonthuis, D.J.; Netz, R.R.; Jungwirth, P. Charge Transfer between Water Molecules As the Possible Origin of the Observed Charging at the Surface of Pure Water. *J. Phys. Chem. Lett.* **2012**, *3*, 107–111.
76. Roncaratti, L.F.; Belpassi, L.; Cappelletti, D.; Pirani, F.; Tarantelli, F. Molecular-Beam Scattering Experiments and Theoretical Calculations Probing Charge Transfer in Weakly Bound Complexes of Water. *J. Phys. Chem. A* **2009**, *113*, 15223–15232.
77. Marenich, A.V.; Cramer, C.J.; Truhlar, D.G. Universal Solvation Model Based on Solute Electron Density and on a Continuum Model of the Solvent Defined by the Bulk Dielectric Constant and Atomic Surface Tensions. *J. Phys. Chem. B* **2009**, *113*, 6378–6396.
78. Martin, F.; Zipse, H. Charge distribution in the water molecule—A comparison of methods. *J. Comput. Chem.* **2005**, *26*, 97–105.

79. Han, B.; Isborn, C.M.; Shi, L. Determining Partial Atomic Charges for Liquid Water: Assessing Electronic Structure and Charge Models. *J. Chem. Theory Comput.* **2021**, *17*, 889–901.
80. Ghosh, S.R.; Debnath, B.; Jana, A.D. Water dimer isomers: Interaction energies and electronic structure. *J. Mol. Model.* **2020**, *26*, 20.
81. Baker, C.M.; MacKerell, A.D., Jr. Polarizability rescaling and atom-based Thole scaling in the CHARMM Drude polarizable force field for ethers. *J. Mol. Model.* **2010**, *16*, 567–576.
82. Bertie, J.E.; Lan, Z. Infrared Intensities of Liquids XX: The Intensity of the OH Stretching Band of Liquid Water Revisited, and the Best Current Values of the Optical Constants of H_2O(l) at 25 °C between 15,000 and 1 cm^{-1}. *Appl. Spectrosc.* **1996**, *50*, 1047–1057.
83. Martinez, L.; Andrade, R.; Birgin, E.G.; Martinez, J.M. Packmol: A package for building initial configurations for molecular dynamics simulations. *J. Comput. Chem.* **2009**, *30*, 2157–2164.
84. Brooks, B.R.; and Brooks, C. L., III.; MacKerell, A.D., Jr.; Nilsson, L.; Petrella, R.J.; Roux, B.; Won, Y.; Archontis, G.; Bartels, C.; Boresch, S.; Caflisch, A.; Caves, L.; Cui, Q.; Dinner, A.R.; Feig, M.; Fischer, S.; Gao, J.; Hodoscek, M.; Im, W.; Kuczera, K.; Lazaridis, T.; Ma, J.; Ovchinnikov, V.; Paci, E.; Pastor, R.W.; Post, C.B.; Pu, J.Z.; Schaefer, M.; Tidor, B.; Venable, R.M.; Woodcock, H.L.; Wu, X.; Yang, W.; York, D.M.; Karplus, M. CHARMM: The biomolecular simulation program. *J. Comput. Chem.* **2009**, *30*, 1545–1614.
85. Liu, D.C.; Nocedal, J. On the limited memory BFGS method for large scale optimization. *Math. Program.* **1989**, *45*, 503–528.
86. Michaud-Agrawal N.; Denning, E.J.; Woolf, T.B.; Beckstein, O. MDAnalysis: A Toolkit for the Analysis of Molecular Dynamics Simulations. *J. Comput. Chem.* **2011**, *32*, 2319-2327.
87. Gowers, R.J.; Linke, M.; Barnoud, J.; Reddy, T.J.E.; Melo, M.N.; Seyler, S.L.; Dotson, D.L.; Domanski, J.; Buchoux, S.; Kenney, I.M.; Beckstein, O. MDAnalysis: A Python package for the rapid analysis of molecular dynamics simulations. *Proceedings of the 15th Python in Science Conference* **2016**, 102–109.
